# In Vivo, In Vitro and In Silico Studies of the Hybrid Compound AA3266, an Opioid Agonist/NK1R Antagonist with Selective Cytotoxicity

**DOI:** 10.3390/ijms21207738

**Published:** 2020-10-19

**Authors:** Joanna Matalińska, Piotr F. J. Lipiński, Piotr Kosson, Katarzyna Kosińska, Aleksandra Misicka

**Affiliations:** 1 Department of Neuropeptides, Mossakowski Medical Research Centre Polish Academy of Sciences, Pawińskiego 5, 02-106 Warsaw, Poland; kkosinska@imdik.pan.pl (K.K.); misicka@chem.uw.edu.pl (A.M.); 2 Toxicology Research Laboratory, Mossakowski Medical Research Centre Polish Academy of Sciences, Pawińskiego 5, 02-106 Warsaw, Poland; pkosson@imdik.pan.pl

**Keywords:** cytotoxicity, fragment molecular orbitals, melanoma, molecular dynamics, morphine, multitarget ligands, NK1 receptor antagonists, opioid, pain, tolerance

## Abstract

AA3266 is a hybrid compound consisting of opioid receptor agonist and neurokinin-1 receptor (NK1R) antagonist pharmacophores. It was designed with the desire to have an analgesic molecule with improved properties and auxiliary anticancer activity. Previously, the compound was found to exhibit high affinity for μ- and δ-opioid receptors, while moderate binding to NK1R. In the presented contribution, we report on a deeper investigation of this hybrid. In vivo, we have established that AA3266 has potent antinociceptive activity in acute pain model, comparable to that of morphine. Desirably, with prolonged administration, our hybrid induces less tolerance than morphine does. AA3266, contrary to morphine, does not cause development of constipation, which is one of the main undesirable effects of opioid use. In vitro, we have confirmed relatively strong cytotoxic activity on a few selected cancer cell lines, similar to or greater than that of a reference NK1R antagonist, aprepitant. Importantly, our compound affects normal cells to smaller extent what makes our compound more selective against cancer cells. In silico methods, including molecular docking, molecular dynamics simulations and fragment molecular orbital calculations, have been used to investigate the interactions of AA3266 with MOR and NK1R. Insights from these will guide structural optimization of opioid/antitachykinin hybrid compounds.

## 1. Introduction

Pain continues to be a major global medical and public health problem. This is especially true of chronic pain which is a co-morbidity associated with musculoskeletal problems, neuropathies, cancers and other diseases. Epidemiologic studies show that as much as 20% of the adult European population experiences chronic pain [1]. 

Among the medicines most often used for treating moderate to severe pain are opioid analgesics, including e.g., morphine and fentanyl. These µ-opioid receptor (MOR) agonists are very effective analgesics, however their long-term administration is associated with the development of tolerance and a need for increasing the dosage. Moreover, these drugs are not devoid of adverse effects that either influence the quality of life (e.g., nausea, vomiting, constipation) or even threaten life (e.g., respiratory depression) [2,3]. Other concerns include the development of dependence and addiction. 

In light of these deficiencies of opioids, a vivid area of investigation in modern medicinal chemistry is the attempt to dissociate the desired analgesic action from the undesired tolerance and adverse effects. One of the approaches towards this problem consists in obtaining multitarget analgesic compounds [4,5]. Substances that combine in one molecule two opioid pharmacophores or an opioid and a non-opioid pharmacophore are able to bind with high affinity to more than one molecular target involved in pain modulation. In principle, this should allow for decreasing the dosage (compared to classical opioid) and in turn lower the probability and the rate of tolerance development. Such compounds are also expected to have less therapy-limiting side-effects. Importantly, additional non-opioid analgesic components could provide efficacy in conditions where classical opioids are not particularly useful, e.g., neuropathies. 

Working in the paradigm of multi-functional analgesics, researchers have investigated molecules designed by joining pharmacophores of various molecular targets and functional activities. The considered combinations are as diverse as MOR agonist/DOR agonist [6,7] MOR agonist/DOR antagonist [8,9], MOR agonist/cholecystokinin CCK2 receptor antagonist [10,11], MOR agonist/ neurotensin agonist [12,13], MOR agonist/ melanocortin-4 antagonist [14], MOR agonist/σ1-receptor antagonist [15,16], MOR agonist/voltage gated calcium channel (VGCC) blocker [17], MOR agonist/cannabinoid-1 receptors agonist [18] or MOR agonist/neuropeptide FF receptor agonist [19].

In yet another type of multitarget analgesics, it is the pharmacophore of neurokinin-1 receptor (NK1R) antagonist that is joined with the opioid pharmacophore [20]. The rationale for this merge lies in the role that Substance P (SP), a tachykinin peptide being the major endogenous ligand for NK1R, plays in the pain modulation. SP acts as an excitatory and pronociceptive neurotransmitter [21]. Moreover, it is thought to have a role in chronic inflammatory pain states by taking part in the development of central sensitization and hyperalgesia. Finally, co-localization of opioid and NK1 receptors was documented in some nerve structures responsible for the transmission of nociception [22]. 

Hence, several research groups synthesized and tested chimeric opioid agonist/NK1R antagonist compounds. Their results are summarized and critically discussed in a review by Kleczkowska et al. [20]. In a recent work, Wtorek et al. [23] corroborated the rationale behind designing such hybrids, showing that a cyclic peptide opioid agonist/linear peptide NK1 antagonist has a significant antinociceptive effect but does not trigger tolerance development.

Adding an antitachykinin component to an opioid analgesic may bring in another potentially favourable characteristic which is anticancer action. The NK1R antagonists (mainly of small-molecule character) have been many times shown to exhibit antitumour (antiproliferative, and antimetastatic) activity [24,25,26,27,28]. This was found both in vitro against numerous human cancer cell lines as well as in vivo in animals xenografted with tumours. Therefore, it could be expected that analgesic compounds with NK1R antagonism in their pharmacodynamics profile might have some auxiliary anticancer activity. As such they could be particularly useful for the treatment of cancer pain. 

**Figure 1:**
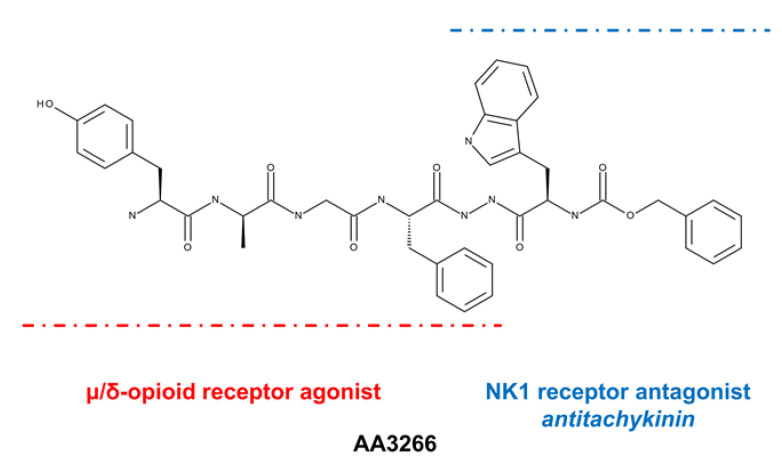
Structure of AA3266.

With this in mind, we synthesized compound AA3266 (Figure 1, disclosed previously [29]) that consists of an enkephalin-like fragment (Tyr-d-Ala-Gly-Phe-) and *N*-carboxybenzyl-d-tryptophan (Z-d-Trp) fragment joined by *N*′-acylhydrazide bridge [17]. The latter fragment can be considered to form the NK1R antagonist pharmacophore.

AA3266 is a strong μ- and δ-opioid agonist with moderate NK1R affinity. Furthermore, we were able to preliminary demonstrate that the compound has a strong inhibitory effect on cellular proliferation in several cancer cell lines while little influence on the proliferation of normal cells [29]. In the presented contribution, we report on a more deeper exploration of pharmacological properties of AA3266. First, the compound was tested in vivo as to its analgesic activity and propensity to develop tolerance and affect the gastrointestinal transit. Second, our hybrid was assayed in vitro as to its cellular effects in cancer and normal cells. Finally, we performed a thorough in silico investigation that gives insight into the interactions that the compound has with its molecular targets.

## 2. Results and Discussion

### 2.1. In Vivo Examination of Antinociceptive Activity, Tolerance and Impact On Gastrointestinal Transit

We have previously found [29] that AA3266 binds strongly to MOR (IC_50_ = 0.06 nM ± 0.01), DOR (IC_50_ = 0.6 nM ± 0.24) and with moderate affinity to human NK1R (Ki = 180.0 nM ± 13.5). Furthermore, in the [^35^S]GTPγS assay, the compound was shown to be a highly potent, full opioid agonist. Prompted by these results, we decided to test in vivo:

(1) the compounds’ antinociceptive activity in an acute pain model,
(2) its influence on tolerance development,
(3) its influence on gastrointestinal transit.

#### 2.1.1. Antinociceptive Activity

**Figure 2:**
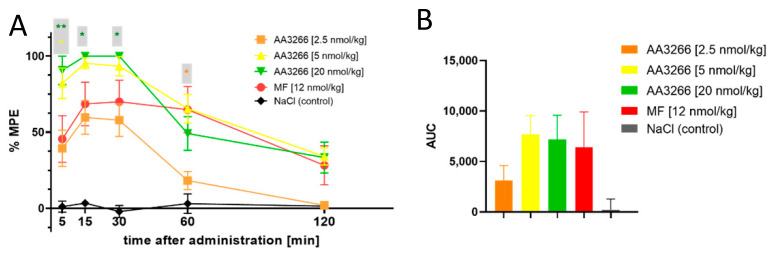
(**A**) Time and dose-dependence of antinociceptive effect (i.t. administration, tail-flick test) of AA3266 compared to positive control (morphine 12 nmol/kg) and negative control (NaCl). Thin bars show standard error of the mean. The asterisks denote statistical significance of the difference between the value found for a particular AA3266 concentration and the positive control (* *p*  ≤  0.05, ** *p*  ≤  0.01). The statistical analysis used is the Fisher’s Least Significant Difference test (planned comparisons; not corrected for multiple comparisons) at significance level α  =  0.05. (**B**) Area under the antinociceptive response curve.

The antinociceptive activity was tested in male Wistar rats in tail-flick test, after the intrathecal (i.t.) administration. NaCl solution was used as a negative control, and a gold standard narcotic analgesic, morphine (MF) (12 nmol/kg dose), was taken as a positive control. The results are presented graphically in Figure 2A as percent of the maximal possible effect (%MPE) plotted against time. Additionally, Figure 2B shows the area under the antinociceptive response curve (AUC) for the test groups.

Consistently with the high opioid receptor affinity and potent activation of the G-protein-mediated signalling, the compound AA3266 exhibits strong antinociceptive effect. The effect is time--dependent, but its dose-dependence cannot be unequivocally stated due to little variance within the effects found for the three tested doses. As little as 2.5 nmol/kg of AA3266 produces a response no different to that exerted by 12 nmol/kg of morphine, if 5, 15 and 30 min post injection timepoints are considered. At 60 min after the administration, for the 2.5 nmol/kg dose, the effect significantly drops in comparison to morphine and it disappears by 120 min. This rapid decrease with time is reflected in a significantly smaller AUC value for 2.5 nmol/kg of AA3266 compared to the positive control. 

Increasing the dose of AA3266 to 5 nmol/kg yields a significantly stronger and quicker analgesic response than in the case of the control morphine. As early as by the 5 min timepoint, it reaches 82% ± 10% MPE. By the 15 min after the injection, the response is 95% ± 4% MPE and after the 30 min timepoint it gradually decreases. Still, at the 120 min timepoint the observed effect is not different than that of the control morphine (34% ± 6% vs. 28% ± 13% for AA3266 and morphine, respectively). 

Even higher dose of AA3266 (20 nmol/kg) neither significantly increases the highest effect nor influences the duration of the antinociceptive activity. Total analgesia (as captured by AUC value) is statistically identical for 5 nmol/kg and 20 nmol/kg of AA3266, as well as for control 12 nmol/kg of morphine.

#### 2.1.2. Tolerance 

**Figure 3:**
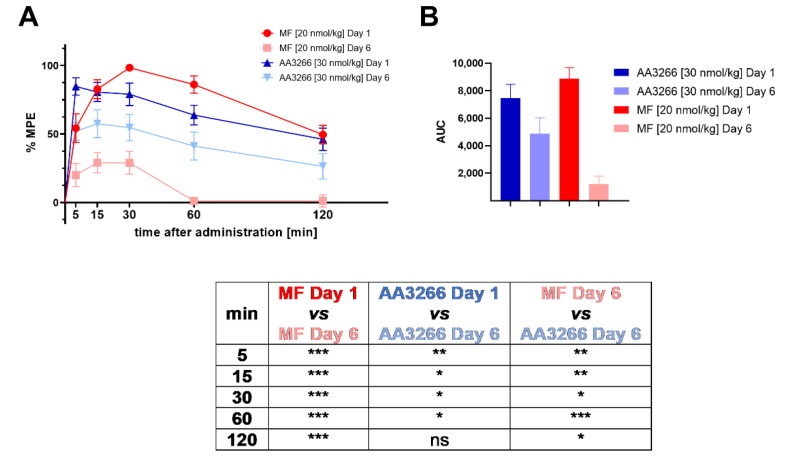
(**A**) Time and dose-dependence of antinociceptive effect (i.t. administration, tail-flick test) of AA3266 (30 nmol/kg) compared to morphine (20 nmol/kg) on Day 1 and on Day 6 after prolonged administration. The results for the negative control (NaCl) were no different than zero in all the time points and so they are omitted from the plot for clarity. Thin bars show standard error of the mean. The results of statistical testing are given in a tabular form below the plots. The asterisks denote statistical significance of the difference between the compared values (* *p*  ≤  0.05, ** *p*  ≤  0.01, *** *p*  ≤  0.001, ns—not significant). The statistical analysis used is the Fisher’s Least Significant Difference test (planned comparisons; not corrected for multiple comparisons) at significance level α  =  0.05. (**B**) Area under the antinociceptive response curve (prolonged administration).

In order to examine whether the rats develop tolerance to AA3266, the compound (in a dose escalated to 30 nmol/kg) was administered intrathecally for six consecutive days. The antinociceptive effect was measured on Day 1 and Day 6 in tail-flick test. Again, NaCl solution was used as a negative control, and morphine was taken as a positive control (in an escalated dose of 20 nmol/kg). The results are presented in Figure 3A as percent of the maximal possible effect (%MPE) plotted against time. The area under the antinociceptive response curve (AUC) for the test groups is shown in Figure 3B.

The experiment revealed that our compound, AA3266, has less propensity to induce tolerance development than the standard opioid analgesic, morphine. What is worth stressing, AA3266 was administered in a higher dose (30 nmol/kg) than morphine was, but still it suffered a smaller reduction of antinociceptive effect after 6 days of administration. 

The response measured for AA3266 (at each time point, Day 6) is on average 65 ± 5% of the values found on Day 1. For morphine, it is only 21 ± 15% of the values found on Day 1. The difference in tolerance development can be seen also in total analgesia. In the case of AA3266, AUC value decreases from 7466 ± 994 (Day 1) to 4859 ± 1171 (Day 6, 65% of AUC on Day 1). For the control morphine, this drop is even more pronounced since the AUC goes from 8882 ± 802 (Day 1) to 1207 ± 585 (Day 6, 14% of AUC on Day 1).

#### 2.1.3. Influence on Gastrointestinal Transit

**Figure 4:**
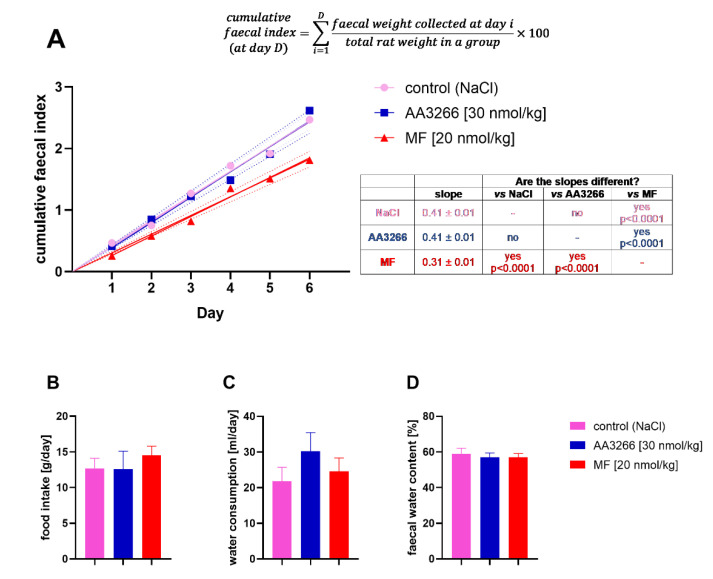
The influence of the prolonged administration of AA3266, MF and NaCl on (**A**) the cumulative faecal index, (**B**) food intake, (**C**) water consumption and (**D**) faecal water content. The line in the (**A**) subplot is a curve of linear relationship between the index and the experiment day. The dotted lines represent 95% confidence intervals of the linear curve. Whether the regression slopes are different for the groups, was tested with the extra sum-of-squares F test. In the subplots (**B**–**D**), the bars represent the mean over the 6 days with the standard error of the mean. For these data (food intake, water consumption and faecal water content), according to the one-way analysis of variance (ANOVA), there is no significant difference between the means (α = 0.05).

The influence on gastrointestinal transit with the prolonged administration of AA3266 was measured by collecting and weighing faecal of the rats used in the tolerance test. The positive and negative control groups consisted of the animals injected with morphine and NaCl. For quantitative comparisons between the groups, we used the cumulative faecal index, that is a cumulative sum of faecal weight multiplied by 100 and divided by the weight of the animals in the group found for a given experimental day and all the days preceding it. The time plot of this value is given in Figure 4A. Additionally, the food intake, water consumption and faecal water content were measured for the experimental groups (Figure 4B–D).

Neither the test (AA3266) nor the positive control (MF) groups differed from the negative control (NaCl) with respect to the food intake, water consumption and the faecal water content (Figure 4B–D). Still, the decrease in the amount of expulsed faeces was clearly observed in the MF group, while our compound, AA3266, was found to have no negative effect on the gastrointestinal function (Figure 4A). The linear curves (forced through the origin) derived for the cumulative faecal index relationship with time (correlation coefficient R~0.99 for all groups) had the slopes 0.41 ± 0.01, 0.41 ± 0.01 and 0.31 ± 0.01 for the AA3266, NaCl and MF groups, respectively. 

#### 2.1.4. Discussion of the In Vivo Results

The data presented above demonstrate that AA3266 has a strong antinociceptive activity in the acute pain model and that the activity is of intensity similar to that of morphine. This is consistent with high opioid affinity of our compound. After 6 days of administration of AA3266 in a high dose, some tolerance develops, but it is significantly smaller than that associated with the administration of morphine. Importantly, AA3266 does not produce constipation (contrary to morphine).

Strong analgesic action and less propensity to trigger tolerance development is likely associated with the presence of antitachykinin component in AA3266. This was the design assumption based on rich data regarding the interactions between opioid receptors and NK1R [22]. Both types of receptors are colocalized in nervous system, in particular in structures involved in nociceptive transmission. Not only is Substance P considered to be a pronociceptive agent, but SP-NK1R activation was shown to be involved in central sensitization to pain. Sustained activation of the opioid system is compensated for by the increased production of pronociceptive factors, including Substance P, as well as their receptors, NK1R among others [30,31]. This in turn may result in decreasing the analgesic efficacy of opioid receptor activation or even in opioid hyperalgesia. Such processes are among the considered mechanisms of opioid tolerance development [32]. Hybrid compounds of the opioid/antitachykinin character should be able to achieve high antinociceptive effect (via the opioid receptors) and to counteract the consequences of the undesired plasticity of the pronociceptive systems (by antagonising the NK1R). Hence, with the reduced/eliminated need for escalating the dosages, compounds of this kind are hoped to give strong analgesia with less tolerance and less adverse effects. 

This line of reasoning was supported by early data that NK1R antagonists (which on their own are not effective analgesics) when co-administered with opioids enhance the antinociceptive activity of the latter. For example, Misterek et al. showed that co-administration of an NK1R antagonist, compound SPA (in 0.25 μg dose), increased the duration of action of an opioid peptide, biphalin [33]. It was furthermore observed that co-administration of NK1R antagonist and opioids inhibited tolerance development upon prolonged opioid administration [34]. 

Beneficial contribution of the antitachykinin fragment to the activity profile of AA3266 is probable also in the light of what was established for a closely related compound AA501 (Tyr-d-Ala-Gly-Phe-NH-NH<-Z-Trp) [35]. This hybrid, which differs from AA3266 only by stereochemistry of the Trp residue, was found to produce effective antinociception in acute and neuropathic pain models. The prolonged administration caused however tolerance whose development rate was greater if Substance P was co-administered, proving that the SP-NK1R system is involved in this process.

Several other potent opioid/antitachykinin hybrids have been already known. Thoroughly evaluated examples are compounds TY005 [36] and TY027 [37] that were designed based on biphalin sequence and the structure of mixed peptide-organic NK1R pharmacophore [38]. These were shown to have strong antinociceptive effects in both acute and neuropathic pain models without propensity to induce tolerance development. 

Other group of hybrids was devised based on Dmt-d-Arg-Phe-Lys-NH_2_ ([Dmt^1^]-DALDA) structure [39] in which a constrained aromatic amino acid was introduced in position 3. Fusing such opioid structure with a few different NK1R antagonist motifs yielded some potent analgesics that were very effective in neuropathic pain models [40,41,42], even though not always more effective than morphine in acute pain [41,42]. For some of these compounds, a tolerance profile similar to that of morphine [43] as well as cross-tolerance with this alkaloid was reported [41], while in one case such cross-tolerance was not found [40].

Recently, Wtorek et al. reported a series of hybrids based on a cyclic opioid peptide and linear peptide NK1 agonist/antagonist fragments [23]. One of these NK1R-antagonist-containing compounds was shown to be effective in acute pain model and not to trigger tolerance development.

The issue of gastrointestinal transit impairment was touched upon only a few times in the context of hybrid opioid/antitachykinin compounds. The aforementioned work by Wtorek et al. demonstrated that their opioid/antitachykinin ligand did not produce constipation [23]. Earlier, Largent-Milnes et al. reported that compound TY027 exerted no influence on gastrointestinal transit [37]. 

### 2.2. Cytotoxicity

#### 2.2.1. In Vitro Assessment of Cellular Pharmacological Effects in Selected Cancer and Normal Cells

**Figure 5:**
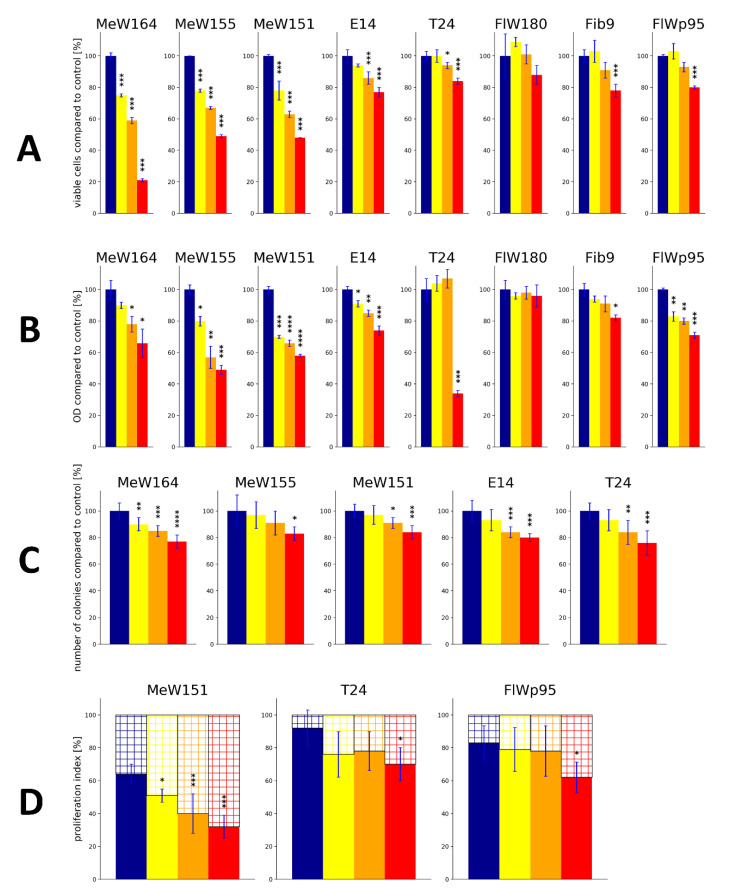
The cellular pharmacological effects of AA3266 in selected cell lines. (**A**) Number of cells counted in haemocytometer (data taken from Ref. [29]) (**B**) Results of the MTT assay. (**C**) Effect on the extent of the colony formation. (**D**) Effect on the expression of the Ki67 protein. The data are expressed as percentage of the values found for the control. Bar colouring corresponds to the concentration of AA3266 (red—100 µM; orange—50 µM; yellow—25 µM; blue—0 µM, control). Cell lines designations given in text. Blue thin bar shows standard deviation. The data come from two independent experiments done in triplicate. The asterisks denote statistical significance of the difference between the given value found for the given concentration and the control (* *p* ≤ 0.05, ** *p* ≤ 0.01, *** *p* ≤ 0.001, **** *p* < 0.0001). The statistical analysis used is the one-way ANOVA with post-hoc Dunnett test at significance level α = 0.05.

In our previous contribution [29] we demonstrated that AA3266 has a strong inhibitory effect on cellular proliferation in several cancer cell lines (Figure 5A). The extent of this effect was similar to or greater than that found for an approved NK1R antagonist, aprepitant. On the other hand, our compound had little or no inhibitory effect on normal cells, while aprepitant exerted some toxicity. In order to corroborate these findings, we have now performed a follow-up examination of the cellular pharmacological effects in cancer and normal cells, using additional assays.

Thus, the effect that AA3266 has on cells was characterized by:

1. direct counting the number of cells in culture after the incubation (reported previously [29]),
2. the MTT assay,
3. measuring the extent of colony formation, and
4. measuring the expression of Ki67 protein (proliferation index).

The results of these tests are presented graphically in Figure 5 as a percent of the assay readout compared to the value found for the control (normalized as 100%, Figure 5A–C) or as a value of proliferation index (Figure 5D).

The MTT assay (Figure 5B) was performed on five human cancer cell lines (melanoma: MeW164, MeW155, MeW151; lung cancer E14 and urinary bladder carcinoma T24) and three human normal cell lines (adult fibroblasts: Fib9 and FlW180; as well as foetal fibroblast FlWp95). In the case of cancer cell lines, AA3266 has a statistically significant effect on the MTT readout in almost all tested concentrations (25 µM, 50 µM, 100 µM), the exception being the T24 cell line. In the melanoma cells, as well as in E14 lung cancer cells, the concentration of 25 µM is able to inhibit cellular proliferation. In the highest concentration (100 µM), the inhibitory effect is present also in the T24 cell line. On average, in the cancer cells, the value found for 100 µM AA3266 reads 62% ± 5% of the control value (if MeW164, MeW155, MeW151 and E14 cells are considered). 

On the contrary, normal cells are much less sensitive to AA3266. The FlW180 line exhibits no drop in MTT readout in any of the concentration tested. In the case of another adult fibroblast cell line, Fib9, it is 100 µM that is required to obtain a statistically significant reduction of optical density. Still, this reduction is not too great since with this concentration the value found is 82% ± 2% of the control value. The most sensitive is the FlWp95 in the case of which all concentrations reduce cellular proliferation to some extent. For this cell line, 100 µM AA3266 gave the MTT readout of 71% ± 2% (compared to the control).

Regarding the colony formation (Figure 5C), its extent was tested in five cancer cell lines (the normal cells did not form colonies in the particular assay conditions). In the case of MeW164, AA3266 suppressed the colony formation in all tested concentrations. In MeW151, E14 and T24 cell lines, the reduction was observed with 50 µM and 100 µM. The least sensitive to AA3266 was MeW155 line. A concentration as high as 100 µM was required for a significant reduction of colony formation. On average, 100 µM of AA3266 reduced the number of colonies to 80% ± 4% of the control value. 

Finally, we determined the impact of AA3266 on the Ki-67 protein expression in three cell lines (2 cancer and 1 normal one, Figure 5D). The Ki-67 protein is a marker of proliferating cells, since it is expressed during all phases of the cell cycle (G_1_, S, G_2_, M), but it is undetectable in the resting state (G_0_ phase) [44]. The Ki-67 proliferation index is the percentage of cells that are found to express this protein (by immunostaining). In this test, AA3266 was shown to potently reduce the proliferation index in MeW151 cell line. Here, a concentration as low as 25 µM impacts the index to a statistically significant extent, and 100 µM reduces the index by 32 percentage points (from 64% ± 6% for control to 32% ± 7%). On the other hand, both urinary carcinoma T24 and foetal fibroblasts FlWp95 were insensitive to 25 µM and 50 µM of AA3266. With the 100 µM concentration, the reduction of the index was 22 and 21 percentage points, for T24 and FlWp95, respectively. 

#### 2.2.2. Discussion of the In Vitro Results

In agreement with our previous work [29] and consistently with affinity for the NK1R receptor, AA3266 was again demonstrated to have a strong inhibitory effect on cellular proliferation in several cancer cell lines. Taking advantage of the fact that we have previously tested aprepitant [45] and compound AWL3020 (hybrid opioid/NK1R ligand) [44] in the very same set of experiments and cell lines, we can now directly compare the action of AA3266 and that of those compounds. Table 1 contains average readout reductions and selectivity factors found in cell counting and MTT assays for 100 µM AA3266, aprepitant and AWL3020 in the tested melanomas, cancers in general (melanomas as well as E14 and T24 cells) and normal cells.

Analysis of these data reveals that AA3266 is more potent than AWL3020 and aprepitant in reducing cell number in melanoma cell lines. On the other hand, when MTT assay is considered, all three compounds have similar activity against melanomas. If all cancer lines are taken for averaging, both the cell counting and MTT assay show that the compounds are equipotent, reaching about ~40% readout reduction compared to the control. 

Importantly, the compounds differ significantly in their action on normal cells. AA3266 is less active against normal cells than AWL3020 and aprepitant are. Moreover, the discussed hybrid exhibits far greater selectivity as reflected by the selectivity indices (all cancers vs. normal cells). In cell counting and MTT assays, these indices read 2.5 and 2.6 for AA3266, whereas for AWL3020 they stand at 1.4 and 1.0. In the case of aprepitant, these selectivity indices are 1.2 and 1.3. Hence, it can be summarized that in this set of experiments and cell lines, AA3266 is selectively cytotoxic, while AWL3020 and aprepitant are not.

That the studied set of cell lines seems to be more resistant to aprepitant than the lines tested by other authors are, we have noted previously [45]. In majority of the reports by other workers, aprepitant was found to exert cytotoxic activity with IC_50_ values of around 20–40 µM and IC_100_ of 40–80 µM [46,47,48,49,50,51]. Moreover, in HEK293 cell lines, it was demonstrated that aprepitant affects normal cells with IC_50_ three times higher than cancer cell lines. In the light of this, evaluating AA3266 in the cell lines used by other groups seems an important next step to confirm its relative potency compared to aprepitant.

So far, the idea to combine opioids and NK1R antagonists to achieve auxiliary anticancer activity has been considered only a few times in the literature. These include our previous contribution on AA3266 [29] and the above mentioned AWL3020 [44]. Recently Dyniewicz et al. reported a series of hybrid compounds in which various opioid sequences were appended (at the C-terminus) with the 3,5-bis(trifluoromethyl)phenyl moiety characteristic for neurokinin-1 antagonists [17]. The compounds turned out to have very low or negligible rat NK1R affinity. Still they showed a diversified range of cytotoxic in vitro activities against MeW155 melanoma cell line (used also in the herein reported contribution), with a few of them being significantly more cytotoxic than aprepitant. A linear correlation between ALogP and cytotoxicity was found suggesting that either non-receptor mediated cytotoxic mechanism is present or a limiting factor for the activity is the ability to cross the cellular membrane. 

Other opioid-based hybrids that were evaluated as to their activity against cancer cells were built of µ-opioid tripeptide sequence and *trans*-1-cinnamylpiperazine moiety [52]. The compounds (high affinity MOR ligands) strongly decreased cell viability in 2D and 3D cell cultures of pancreatic cancer. The compounds were however not selective and normal cells were sensitive to their action, too.

The results of Ki-67 proliferation index testing show that AA3266 decreases the potential of cells to proliferate, the effect being the strongest in the melanoma MeW151 cells and relatively weak in urinary bladder carcinoma T24 and normal FlWp95 cells. This may suggest that the cellular effects seen for AA3266 are (at least partially) associated with decreasing division of cells. Regarding other hybrid compounds with the NK1R pharmacophore, it is only the aforementioned AWL3020 (a weak NK1R binder), for which the Ki-67 testing have been reported [44] with qualitatively similar results as in the current study.

In general, NK1R antagonists are thought to exert their anticancer activity by inducing cell apoptosis in an NK1R-dependent manner [47]. The signaling pathways and mechanisms underlying this have been recently summarized [25]. Worth mentioning here, in terms of the influence on the cell cycle, is that aprepitant was found to promote G2/M-phase cell-cycle arrest in breast cancer cells [53] and esophageal squamous cell carcinoma [54]. Aprepitant alone (and even more potently in synergistic combination with cytosine arabinoside) induced cell cycle arrest in G0/G1 phase in acute myeloid leukaemia HL60 cell line [55]. With respect to the Ki-67 index, in vivo, administration of aprepitant to nude mice xenografted with HuH6 cell line (human hepatoblastoma) decreased the Ki-67 index value by about a third compared to the placebo control [56]. Similarly, the reduction was observed in an animal model created with human melanoma A-375 cell lines [57]. These and other rich data on the effects of aprepitant on cancer cells [25] point to the possible directions of investigation that may be undertaken to fully understand the antiproliferative action of AA3266.

### 2.3. In Sillico Examination of Receptor-Ligand Interactions

In order to understand the structural basis of the interactions between AA3266 and its molecular target (MOR and NK1R), we modelled the complexes of our compound with these receptors. The modelling workflow consisted of molecular docking, molecular dynamics (MD) simulations and fragment molecular orbital (FMO) calculations. The former two give prediction as to the geometry of the complex and its dynamic behaviour, while the latter provides detailed insight into the interaction energetics by the means of pair interaction energy decomposition analysis (PIEDA).

#### 2.3.1. µ-Opioid Receptor

The starting point for modelling of AA3266 complex with the µ-opioid receptor was the experimental structure of DAMGO bound to the MOR (PDB accession code: 6DDF [58]). DAMGO (Tyr-d-Ala-Gly-*N*Me-Phe-Gly-ol) is an enkephalin-derived, selective and potent µ-opioid agonist. The opioid part of AA3266 (Tyr-d-Ala-Gly-Phe-) is closely related to DAMGO, thus it is reasonable to assume that the binding mode of our compound would be similar to the one of this peptide. Therefore, an initial guess at the binding pose of AA3266 and at MOR was done by manually replacing the Gly^5^-ol in DAMGO/MOR complex by -NH-NH<-Z-d-Trp fragment and removing the *N*-methyl in the fourth position. The molecule was then subject to optimization by the local search docking in AutoDock 4.2.6 [59]. This procedure yielded poses in which the opioid part did not deviate significantly from the position of DAMGO in 6DDF, while the NK1 part assumed a few positions close to the extracellular outlet of the binding site. The best scored solution was chosen for MD simulations (7 repetitions of 150 ns length production, see Figure S1 in Supplementary Materials for RMSD plots). 

Visual analysis of the trajectories and a look at RMSD plots (Figures S2 and S3) revealed that the binding pose of the opioid part of AA3266 was rather stable throughout the simulations. On the contrary, the structural elements of the antitachykinin pharmacophore assumed a few different binding poses and exhibited mobility in the simulations (Figure S3). For getting a more precise description, the trajectories (with t > 10 ns) were concatenated, superposed to a common reference structure and clustered with respect to position of the Tyr^1^ amine, Tyr^1^ side chain, Phe^4^ side chain, the opioid part as a whole, d-Trp, carboxybenzyl (Z) and the antitachykinin part as a whole. Hereafter, the clusters (or representative structures belonging to the clusters) will be designated by the receptor abbreviation and a number (if opioid part is discussed) or a letter (if the -NH-NH<-Z-d-Trp part is discussed), e.g., MOR-1, NK1-1 or MOR-a, NK1-a etc.

**Figure 6:**
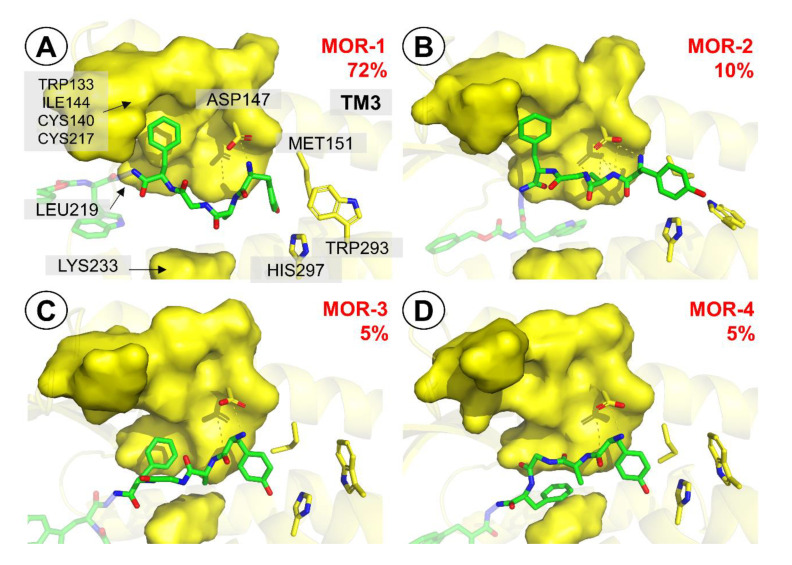
Representative structures for the clusters from MD simulations of AA3266 in the µOR binding site. Focus on the opioid part. (**A**) **MOR-1** cluster, (**B**) **MOR-2** cluster, (**C**) **MOR-3** cluster, (**D**) **MOR-4** cluster. Only a few receptor (yellow) side chains and helices are shown for clarity. The surface covers a few side chains from TM3, ECL2, ECL3 and TM5 important for binding. The red number is the cluster population.

When the opioid fragment is considered, the analysis (at 1.4 Å resolution) reveals 4 clusters. The most populated pose (**MOR-1**, 72%, Figure 6A) closely resembles the DAMGO pose in the 6DDF structure (Figure S4). The complex is stabilized by the canonical interaction Tyr^1^ protonated amine with Asp147 side chain (Figure S5). The phenol group locates closely to His297, and in some simulations there appears a direct H-bond between the group and the histidine ring, while in the others the interaction is mediated by water molecules (Figure S6). The Phe^4^ side chain is located in a hydrophobic subpocket formed by a few residues of transmembrane helix 3 (TM3) and extracellular loops 2 and 3. The contacts in this part include interactions with side chains of Trp133, Cys140, Val143, Ile144, Cys217.

The remaining three clusters are significantly less populated. **MOR-2** pose (Figure 6B) is found in about 10% of simulation time. The pose differs with respect to Tyr^1^ interactions. The side chain protrudes deeper towards the receptor interior. This is associated with a distinctive arrangement of the interactions with Asp147. It is not only the protonated amine of Tyr^1^ that interacts, but also amide hydrogen of D-Ala^2^ is involved. Clusters **MOR-3** and **MOR-4** (Figure 6C,D) are both populated by about 5% of the considered simulation times. Their characteristic feature is different positioning of Phe^4^ side chain. In **MOR-3**, the Phe^4^ aromatic ring locates by the Leu219 side-chain, while in **MOR-4** the peptide rotates so that the Phe^4^ aromatic ring interacts with Lys233.

FMO PIEDA analysis (at the FMO-MP2/6-31G*/PCM level, Table 2) of the discussed binding poses reveals that **MOR-1** and **MOR-4** have the strongest interactions (−226.3 and −223.4 kcal/mol, respectively), while the remaining two poses are weaker by about a quarter. In all cases however, interactions of the opioid fragment are predominantly polar (polar interactions contribution to the energy %E_es+ct_ in the range of 82–88%; see Methods for definition of %E_es+ct_) and the greatest contribution stems from the interaction of Tyr^1^ with Asp147. This is in fact not unexpected given the known importance of the canonical ionic interaction between opioid’s protonable amine and the side chain of Asp147 [60,61,62]. A surprisingly small interaction energy contribution is related to Phe^4^_._ In the **MOR-1** binding pose in which aromatic ring of this side chain is close to the experimental position found for the respective residue in DAMGO/MOR complex, the calculated contribution amounts to only −10 kcal/mol in **MOR-1**. Interestingly, locating this ring closer to Lys233 (**MOR-4**) is associated with a large increase of the interaction energy contribution (to −32.8 kcal/mol) that seems to compensate a decrease in Tyr^1^—Asp147 interaction (due to change in mutual arrangement of amine and acid, Figure 6D), making **MOR-4** equienergetic to **MOR-1**. This may indicate that targeting the Lys233 and the residues around it by nonpolar fragments could provide energy gains in novel ligands. 

**Figure 7:**
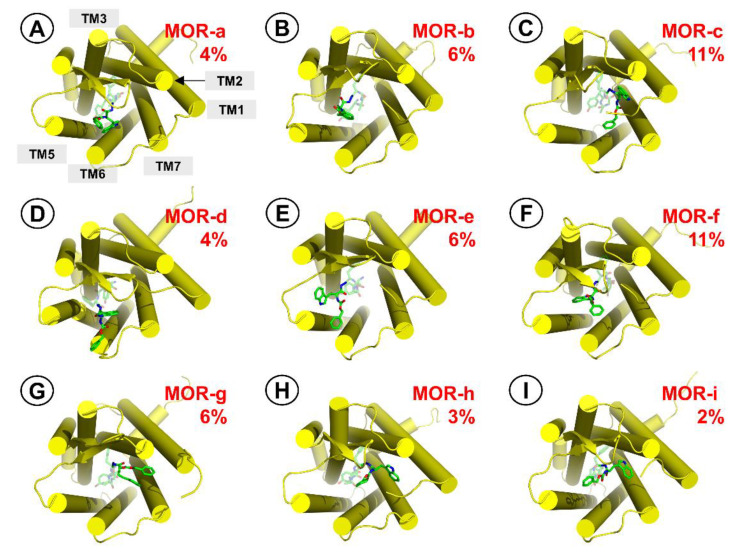
Representative structures for the clusters from MD simulations of AA3266 in the MOR binding site. Focus on the antitachykinin part. (**A**) **MOR-a** cluster, (**B**) **MOR-b** cluster, (**C**) **MOR-c** cluster, (**D**) **MOR-d** cluster, (**E**) **MOR-e** cluster, (**F**) **MOR-f** cluster, (**G**) **MOR-g** cluster, (**H**) **MOR-h** cluster, (**I**) **MOR-i** cluster. Receptor is represented as yellow cylinders (transmembrane helices, TM). The ligand is shown as green sticks. The red number is the cluster population.

If the -NH-NH<-Z-d-Trp fragment is considered, clustering finds 9 relatively stable groups. They are presented graphically in Figure 7 and their intermolecular contacts are summarized in Table 3. The fragment occupies a few distinct positions, and many snapshots in the concatenated set represent transient states that do not fit in any of these significantly populated clusters. Majority of the observed contacts are hydrophobic. In some cases, the hydrazide moiety is involved in polar interactions with the receptor.

The FMO PIEDA interaction energies found for -NH-NH<-Z-d-Trp part (Table 4) are on average −64.7 kcal/mol (range: −38.4 to −92.7 kcal/mol). In comparison with the opioid part, the polar contribution to the interaction energies is smaller but still dominating (%E_es+ct_ in the range of 58–75%). 

An interesting cluster is **MOR-b** (Figure S7). In this one the fragment twists so that the D-Trp ring is directed towards the binding pocket interior, staying close to D-Ala^2^ side chain and Lys233. That such a positioning is fairly stable calls to mind the fact that there is a plethora of potent opioid peptides in which side-chain-to-side-chain mode of cyclization between positions 2 and 5 is present [63,64]. A binding pose analogous to **MOR-b** could explain the affinity of such peptides. Furthermore, in the very case of AA3266, this suggests also means to design cyclic counterparts of this compound. All the more that again, as in the case of **MOR-4**, interaction of the aromatic ring with Lys233 seems favourable. 

#### 2.3.2. NK1 Receptor

In order to model AA3266 complex with the NK1 receptor, the acylated Ac-NH-NH<-Z-d-Trp fragment was docked to the 6HLL structure [65] with AutoDock 4.2.6 [59]. Docking predicted that the fragment goes deep in the binding pocket with d-Trp close to Pro112 and Met297 and the ring of the carboxybenzyl moiety close to His197 and Phe268, while the Ac-NH-NH would be directed to the middle part of the binding pocket. The best scored solution was used for building-in the opioid fragment in a few possible ways. These complexes were then subject to optimization by the local search docking. The best scored solution was chosen for MD simulations (7 repetitions of 150 ns length production, RMSD plots in Figure S8). 

**Figure 8:**
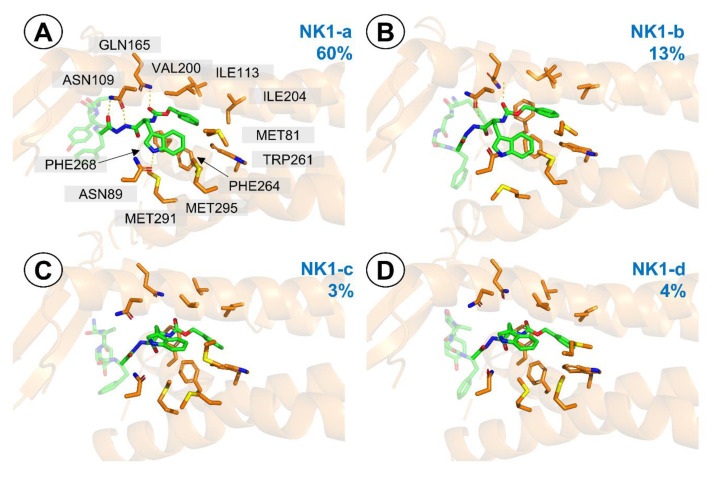
Representative structures for the clusters from MD simulations of AA3266 in the NK1R binding site. Focus on the -NH-NH<-Z-D-Trp part. (**A**) **NK1-a** cluster, (**B**) **NK1-b** cluster, (**C**) **NK1-c** cluster, (**D**) **NK1-d** cluster. Only a few receptor (orange) side chains and helices are shown for clarity. The blue number is the cluster population.

Contrarily to the simulations with µOR (but in some sense, analogically), it is the antitachykinin part that stayed stable during the simulations, while the opioid fragment assumed a few different binding poses (Figures S9 and S10). Clustering (at 0.8 Å resolution) reveals 4 clusters (Figure 8) with respect to the -NH-NH<-Z-d-Trp part, which are nevertheless so similar that by 1.0 Å resolution, one large cluster (~99% of population) appears.

In the **NK1-a** binding pose (60% population, Figure 8A), ring of the carboxybenzyl moiety is wedged between Phe264, Phe268, Trp261, I204, V200. The carbamate carbonyl is involved in an H-bond with Gln165 (Figure S11). d-Trp side chain goes close to Met295, Met291, Met81 and Ile113 and its indole N-H is predicted to be involved in H-bonding with Asn89. Further polar interactions include double H-bonding arrangement between Asn109 and NH/C=O of the *N*′-acylhydrazide element (Figures S12 and S13).

In the **NK1-b** (13% population, Figure 8b), the ring of the carboxybenzyl moiety protrudes deeper towards Val116 and Trp261. d-Trp indole ring is not involved in H-bonding, and the hydrazide has only one H-bond to Asn109. Still, the Gln165 ··· carbamate carbonyl interaction is present. The other two clusters (**NK1-c** and **NK1-d**, populated by 4 and 3%, respectively, Figure 8c,d) have also a deeper positioning of the aromatic ring of Z group and no polar interactions.

FMO PIEDA analysis (Table 5) reveals that the -NH-NH<-Z-d-Trp part enjoys the strongest interaction in the most populated **NK1-a** mode (−78.8 kcal/mol). The remaining modes have worse interaction energies, being −68.7 kcal/mol, −58.2 kcal/mol and −49.8 kcal/mol, in the case of **NK1-b**, **NK1-c** and **NK1-d**, respectively. The interactions of this part are to a large extent hydrophobic (%E_es+ct_ in the range of 43–63%), but presence of the mentioned hydrogen bonds in **NK1-a** shifts this balance in favour of polar character (%E_es+ct_ = 63%). 

**Figure 9:**
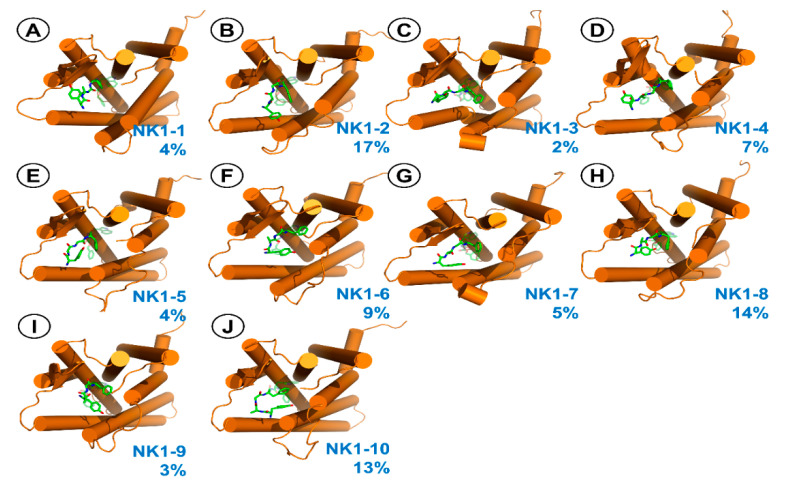
Representative structures for the clusters from MD simulations of AA3266 in the NK1R binding site. Focus on the Tyr-d-Ala-Gly-Phe-fragment. (**A**) **NK1-1** cluster, (**B**) **NK1-2** cluster, (**C**) **NK1-3** cluster, (**D**) **NK1-4** cluster, (**E**) **NK1-5** cluster, (**F**) **NK1-6** cluster, (**G**) **NK1-7** cluster, (**H**) **NK1-8** cluster, (**I**) **NK1-9** cluster, (**J**) **NK1-10** cluster. Receptor is represented as orange cylinders (transmembrane helices, TM). The ligand is shown as green sticks. The blue number is the cluster population.

For the Tyr-d-Ala-Gly-Phe-fragment, 10 clusters (at 1.4 Å resolution, depicted in Figure 9 and described in Table 6) are found. In all these poses, the opioid chain sticks to ECL2. In majority of them, there is an interaction between Tyr^1^ protonated amine and Glu193 (Figure S14). Regarding the position of Phe^4^, the side chain is located in the vicinity of receptor residues such as Tyr92, Val179, Phe267 or Tyr287.

Interestingly, FMO PIEDA analysis (Table 7) predicts that the interactions of the opioid part are stronger than those of the -NH-NH<-Z-d-Trp moiety. The energies found are also much more diversified. The calculated PIEtotal varies between −69.2 kcal/mol and −208.5 kcal/mol, and in most cases is more negative than −100 kcal/mol. In all binding modes, polar character dominates (%E_es+ct_ in the range of 71–91%). This is due to a large contribution of Tyr^1^ amine ⋯⋯ Glu193 interaction. 

**Figure 10:**
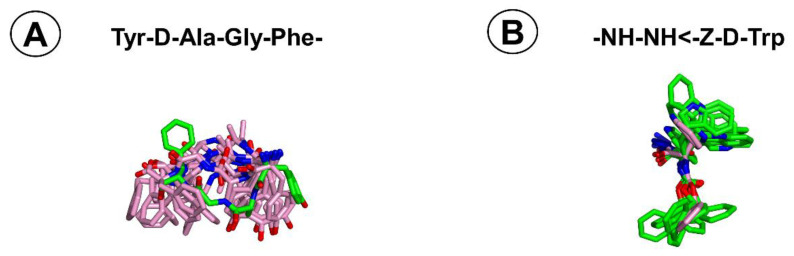
Superposition of the AA3266 conformations found in simulations with MOR and NK1R (**A**) Focus on the Tyr-d-Ala-Gly-Phe-fragment, (**B**) Focus on the -NH-NH-Z-d-Trp fragment. Green sticks represent the structures from simulations with MOR, and pink sticks represent the structures from simulations with NK1R. The reference structures are **MOR-1** for (**A**) and **NK1-a** for (**B**) as the most populated clusters at the respective receptors.

Superposition of the AA3266 structure found in simulations with MOR and NK1R (Figure 10) shows that the Tyr-d-Ala-Gly-Phe-fragment adopts different bioactive conformations at both receptors. Most importantly, mutual orientations of Phe^4^ and Tyr^1^ rings are disparate. In MOR, the rings point in opposite directions (like “up” and “down”), while in NK1R they are on the same side (pointing “down”). Numerically, this is reflected e.g., in distance between CZ atoms of Tyr^1^ and Phe^4^. In **MOR-1** binding pose, the distance is 13.0 Å, whereas in clusters **NK1-1** to **NK1-10** it is 7.4 ± 2.3 Å, on average.

If -NH-NH<-Z-d-Trp superposition is inspected, the conformational difference is not that large. In some instances, the mutual positioning of the aromatic rings in the fragment in poses found in simulations with MOR are identical or very close to **NK1-a**. In others, the difference is associated with a different rotameric state of d-Trp side chain.

#### 2.3.3. Discussion of the In Silico Results

The reported modelling results provide a probable description of the interactions between AA3266 and its molecular targets. At MOR, the most populated cluster (with respect to the positioning of the opioid part) closely resembles the experimental positioning of DAMGO in this receptor. Similar binding poses were also proposed based on molecular modelling results for the opioid fragment of other opioid/antitachykinin hybrids [17] or some Tyr-d-Ala-Phe-Phe (TAPP) derivatives [66]. Aromatic rings of BU72 (experiment [67]), fentanyl (modelling [68]) cyclic opioid derivatives (modelling [69]), or linear peptide opioids (modelling [70]) were found to be located similarly as the Phe^4^ ring in **MOR-1** pose. For the -NH-NH-Z-d-Trp fragment, a few quite distinct binding poses are found at MOR.

At NK1R, the *N*-carboxybenzyl-d-tryptophan moiety occupies the deep portion of the binding pocket, forming interactions analogous to ones found experimentally for small molecular NK1R antagonists [65,71,72]. The opioid fragment extends towards the extracellular outlet of the receptor and the simulations suggest some mobility of this fragment, however an interaction between Tyr^1^ amine and Glu193 is present for a great part of the collected trajectories. Notably, a polar interaction between Glu193 and small molecular ligand was also found in the experimental structure of aprepitant/NK1R complex [65] but SAR data suggest that it is not indispensable to high-affinity binding since the modification [73] or functionalization [74] of the interacting triazolinone ring results in relatively moderate affinity decreases. Moreover, E193A mutation has virtually no effect on aprepitant’s affinity.

Whether multiplicity of the AA3266 poses for the C-terminal fragment at MOR and the N-terminal fragment at NK1R reflects a real residual mobility or is an artefact associated with short sampling times, is hard to conclude without further calculations. Nevertheless, such mobility in these parts cannot be excluded as there are several experimental and computational hints in its favour. 

In one of the experimental structures of the delta-opioid receptor (PDB: 4RWA [75]), electron density of a C-terminal residue (located by the extracellular entry to the binding pocket) suggests a dual conformation. Molecular dynamics simulations of DAMGO in 6DDF structure suggest residual mobility of the C-terminal ‘tail’ [58]. Mixed experimental and computational approach suggested mobility of the C-terminal part in dynorphin bound to kappa-opioid receptor [76]. Interestingly, in that contribution, it is also the N-terminus that was found to be quite flexible. In our work, a corresponding finding is that even though the **MOR-1** cluster (with ‘canonical’ Tyr^1^ position) dominates in the trajectories, noticeable populations are found for slightly different arrangements, including one with a more deep positioning of the tyrosine ring (**MOR-2**). Notably, suchlike positionings of Tyr^1^ were also found in the mentioned dynorphine-KOR complexes [76]. Recently, similar deeper protrusion was proposed for fentanyl [77]. Regarding the NK1R, Hanssen et al. observed computationally that N-terminal portion of Substance P was not well constrained when the ligand was in complex with NK1R, and so multiple conformations are possible [78]. This putative high mobility would fit the experiment observation that multiple sites on the N-terminal tail of NK1R photocross-link to bound SP. 

FMO PIEDA analysis of the studied complexes gives insights into energetics of the interactions. The interaction energy of AA3266 with MOR is dominated by the contribution of the opioid part. Still, in some cases the input of the -NH-NH<-Z-d-Trp could make up to 1/4 of the total energy (e.g., in the case where opioid part contribution would equal to ~−200 kcal/mol and that of the C-terminal fragment would be ~60 kcal/mol). This agrees with the intuition that in multifunctional compounds it is the pharmacophore of a given receptor that would contribute most to the strength of the interactions. 

Counterintuitive seems the finding that for AA3266/NK1R complexes the largest contribution to the interaction energy would come from the opioid part. For clarity, let us note here that the interaction energy is not the binding energy. It describes the strength of interactions in a particular configuration, but omits important factors contributing to binding, e.g., conformational entropy of the interacting partners. Conformational entropy appears most important in case of molecules so flexible as peptides, all the more where multiple binding poses are possible. The FMO findings for AA3266/NK1R complex might suggest that there is a potential for improving NK1R affinity of opioid/antitachykinin hybrids in rigidifying the structure of the opioid part that would reduce the entropic penalty and make the Tyr^1^ and Glu193 interaction more relevant for binding energy. On the other hand, such an endeavour could be complicated by the fact that Glu193 is quite exposed to the solvent and so this contact may finally be weaker than expected. 

An important consideration in the design of multifunctional compounds is whether joining two pharmacophores would not hamper interactions at one/both individual targets. Furthermore, in case of flexible molecules, it seems desirable that bioactive conformations at both targets are similar and are low in energy. Molecular modelling of AA3266 with MOR and NK1R suggests that with respect to the -NH-NH-Z-d-Trp part (which is core of the NK1R pharmacophore), the conformations at both receptors are quite similar (Figure 10). On the contrary, the opioid fragment at the NK1R binding site is predicted to assume conformations different than the dominating one at MOR. This is another information that will guide the optimization of AA3266 structure.

To our knowledge, this is the first published attempt to study binding of hybrid opioid/antitachykinin peptides with molecular modelling methods at two receptors. Whether the findings and conclusions reported here (flexibility of the additional pharmacophore and different conformations of the opioid part in both receptors) hold for other hybrids of this type, will be an interesting issue for further research.

## 3. Materials and Methods 

### 3.1. Chemistry

The compound AA3266 (NH_2_-Tyr-d-Ala-Gly-Phe-NH-NH<-Z-d-Trp was obtained based on the procedure of peptide synthesis in solution using *N*,*N*′-dicyclohexylcarbodiimide (DCC) and N-hydroxysuccinimide (HOSu) strategy, with addition of 1,1,3,3-tetramethylguanidine (TMG), in dimethylformamide (DMF) as described previously [79]. In brief, after assembling the Boc-protected tetrapeptide sequence, the peptide acid was coupled to Z-Trp-NH-NH_2_ and the Boc-protection was removed. 

The crude product was purified using preparative RP-HPLC. The purity of the product was analysed by analytical HPLC with gradient of 3–97% phase B in 31 min (phase A: 0.05% aq. FA, phase B: ACN + 0.05% FA, total flow 1.2 mL/min, column Jupiter^®^ 4 µm, 250 × 4.6 mm, Proteo 90 Å, UV detection at λ = 210 nm, 254 nm and 280 nm and it was found to be greater than 95%. The mass of the compound was confirmed by ESI-MS (ESI-MS ion found *m*/*z* [M + H]^+^: 791.30 calculated [M + H]^+^: 791.35; t_R_ = 10.20 min).

### 3.2. In Vivo Examination of Antinociceptive Activity, Tolerance Development and the Influence on Gastrointestinal Function

The rats (adult male Wistar, weighing 200–250 g, 10 animals for each group) were prepared for the intrathecal administration (i.t.) by a method described previously in detail [44]. All housing and experiments were conducted in accordance with the Polish Act of 21 January 2005 on Experiments on Animals (Journal of Laws No 33 of the Republic of Poland, item 289 as amended). The animal experiments were approved by the IV Local Ethics Committee for Experiments on Animals in Warsaw, Poland (permission no.: 46/2013) and by the I Local Ethics Committee for Experiments on Animals in Warsaw, Poland (permission no.: 519/2018). The experimenters exercised all efforts to minimize the number of animals used and their suffering.

The antinociceptive activity of AA3266 and morphine (MF) was measured by a tail-flick test, as described previously [44]. In brief, the compounds were dissolved in a small amount of DMSO and the saline was added to obtain a required stock concentration. The compounds or a saline solution (Polfa, Warszawa, Poland) were administered via a catheter in a total volume not exceeding 8 µL.

The activity was quantified by using the Plantar Test and Tail Flick Analgesia Meter apparatus (IITC Life Science Inc., Woodland Hills, CA, USA). A radiant heat beam was projected onto the dorsal side of the rat tails and the latency time before the tail withdrawal was measured by a built-in timer.

The measurements were done before the administration (predrug latency), as well as 5, 15, 30, 60 and 120 min thereafter. Each measurement was executed in triplicate with minor changes as to the place where the beam fell. 

The effect was quantified by a maximum possible effect value (%MPE) calculated according to the Brady and Holtzmann formula [80]:

(1) $$\%\;MPE\;=\;100\;\%\;*\;\frac{\mathrm{postdrug}\;\mathrm{latency}-predrug\;latency}{maximum\;latency\;\left(7s\right)\;-\;predrug\;latency}$$

In the first set of experiments, three doses of AA3266 were used (2.5 nmol/kg, 5 nmol/kg and 20 nmol/kg) while for morphine it is 12 nmol/kg that was administered. The negative control was saline solution. 

Having confirmed the activity of AA3266, a second set of experiments was designed and executed, in order to capture the possible development of the tolerance. Here, the compounds or saline were administered for 6 days, once daily. The doses were escalated to 30 nmol/kg (AA3266) and 20 nmol/kg (MF). The antinociceptive effect was measured on Day 1 and Day 6. 

Additionally, the mass of the animals, their food intake and water consumption, as well as the weight of their faeces were monitored. In order to measure the latter, the rats were daily placed in separate clean cages and administered the drug, whereafter their faeces were being collected for 120 min (immediately after expulsion) and put in a closed tube. Then, the faeces were weighed, dried for 24 h at 60 °C and weighed again. It served along with the results of the first weighing to determine the % content of the faecal water.

For assessing constipation, we used the cumulative faecal index. For each day, the faecal index was calculated. It was the faecal weight found on that day for groups multiplied by 100 and divided by the mass of all rats in a given group. The cumulative index value was then obtained by adding the faecal index calculated for a particular day and the indices for all preceding days. 

Linear relationships between the experiment time and the cumulative index were derived (R^2^~0.99, curves forced through the origin). Slopes of the curves were used for comparative purposes.

Statistical analyses and plotting were done in GraphPad Prism [81].

### 3.3. Cell Cultures

In the study, five human cancer cell lines (melanoma: MeW151, MeW155, MeW164; lung cancer: E14 and urinary bladder carcinoma: T24) as well as three human normal cell lines (adult fibroblast lines: Fib9 and FlW180; and foetal fibroblast line: FlWp95) were used. They were obtained from the institutional cell bank at the Maria Sklodowska-Curie Memorial Institute and Oncology in Warsaw, Poland. For culturing the cells, Eagle’s 1959 MEM medium (Biomed, Lublin, Poland) was used. The medium was supplemented with 10% foetal calf serum (Invitrogen, Waltham, MA, USA). The cells were kept at 37 °C, in humidified atmosphere containing 5% CO_2_.

### 3.4. Cellular Assays

The effect that AA3266 exerts on cells was tested in vitro with respect to three endpoints. These were:

(1) Influence on cell viability as measured by the MTT assay (eight cell lines).
(2) Influence on the ability to form colonies (five cancer cell lines).
(3) Influence on the Ki67 proliferation index (three cell lines).

Data related to yet another endpoint, that is influence on the number of cells after 4 or 7 days incubation, come from the previous report [29]. The tests were performed following the procedures previously described [44]. In brief, the cells were incubated in the presence of three concentrations of AA3266 (25 µM, 50 µM and 100 µM, in separate wells/dishes). The incubation time was 24 h (MTT assay) 7 days (colony assay) or 4 days (Ki67 assay). The number of the cells seeded was either 5000 cells per well (MTT and Ki67 assays) or 100 cells per dish (colony assay). With the incubation having been finished, additional steps if necessary were executed (see ref. [44] for details) and the readout followed. The measured values were optical density (MTT assay), number of colonies (colony formation assay) or number of cells expressing the Ki67 protein. The negative control were cells incubated and treated as required by each assay procedure, but without the presence of AA3266. 

The presented results of each assay come from two independent experiments done in triplicate, and they are means with standard deviations. They were analysed with the one-way ANOVA test with post-hoc Dunnett test at significance level α = 0.05.

In the MTT and colony assays, the results were normalized so that the value found for the control be 100%. In the Ki67 assay, the number of cells in the field was set as 100%. The proliferation index was the percent of cells exhibiting Ki-67 expression in randomly selected populations. In each Ki67 experiment, the counting was performed on 10 views.

### 3.5. Molecular Docking

Binding poses of AA3266 at MOR and NK1R were obtained by using AutoDock 4.2.6 [59]. In the case of MOR, for the initial guess at the AA3266/MOR complex geometry, experimental position of DAMGO at MOR (as found in 6DDF [58]) was used. To this aim, Gly^5^-ol of DAMGO was manually replaced by -NH-NH-Z-d-Trp fragment and the *N*-methyl in the fourth position was removed. The molecule was then subject to optimization by the local search docking in AutoDock 4.2.6 [59]. 

In the case of NK1R, Ac-NH-NH<-Z-d-Trp was docked into 6HLL structure [65] with AutoDock 4.2.6 [59]. In the best scored pose, the acyl group was removed and the Tyr-d-Ala-Gly-Phe-fragment was built in in a few possible conformations. The molecule was then subject to optimization by the local search docking in AutoDock 4.2.6 [59]. 

The ligands and the protein structure were processed in AutoDock Tools 4 [59] with standard routines. For both normal and local docking, full ligand flexibility (except for amide bonds) was allowed while the receptor was set as rigid.

The receptor structures (6DDF [59]. and 6HLL [65], for MOR and NK1R, respectively) used for docking and further modelling were refined ones (as provided by the GPCRdb service [82]). In this way, mutated residues were replaced with native ones, and side chains missing in the original PDB structures were supplemented. Before being used for docking, the receptor coordinates were transformed so that they matched the coordinates of corresponding models in OPM database [83]. This facilitated embedding in lipid membrane for the purposes of molecular dynamics.

The docking boxes were set around the experimental position of CP-99,994 in 6HLL or DAMGO in 6DDF and extended towards the extracellular outlet of the binding sites so as to ensure enough space for docking of AA3266. The grids were calculated with AutoGrid 4 [59].

For global docking, default AutoDock parameters were used. For local searches, we used the following settings: 300 individuals in population, 500 iterations of the Solis-Wets local search, the *sw*_*rho* parameter of the local search space set to 20.0, and 1000 local search runs. The calculations were repeated 50 times. The local docking results were clustered, and structures from the best scored clusters were taken for further analyses. 

### 3.6. Molecular Dynamics

In preparation for molecular dynamics simulations, the receptor-ligand systems were embedded in POPC membrane (145 lipid molecules) and solvated with TIP3P water (about 13,000 water molecules, TIP3P) with the help of the CHARMM-GUI service [84]. Na^+^ and Cl^−^ ions (0.154 M concentration) were added, too. CHARMM 36 force field was used for the proteins, lipids, water, and ions. AA3266 was modelled using CHARMM CGenFF [85].

The simulations were run in GROMACS 5.1.2 [86]. The complexes were minimized and equilibrated, whereafter the production followed (NPT ensemble, 303.15 K, integration step = 2 fs, cut-off scheme Verlet, Nose-Hoover thermostat, Parrinello–Rahman barostat, LINCS H-bonds constraints). For each system, 7 runs of 150 ns production length were obtained.

For analysing conformational behaviour of the complexes, the trajectories for each receptor were concatenated and superposed on a common reference snapshot. The superposition was based on backbone atoms of the helical part of the receptors. The root-mean-square deviations of the atomic positions of the protein (in helical part), the ligand, or ligand’s parts were monitored over simulations times. The same were a few interesting distances corresponding to the important interactions between the ligand and the binding site. 

The trajectories were clustered (using built-in GROMACS tools) with respect to the position of the ligand or its subfragments, at a few different resolutions to ensure a good trade-off between the number of clusters (so that they are amenable to analysis) and the desire to capture most of the relevant conformational diversity. The cluster populations were calculated by dividing number of snapshots belonging to a particular cluster and a total number of snapshots subjected to clustering. 

The molecular graphics were prepared in open-source PyMol [87].

### 3.7. Fragment Molecular Orbitals Calculations

Fragment Molecular Orbitals (FMO) calculations were performed on structures representative for the most significant clusters in the MD trajectories. The computations were done in General Atomic and Molecular Electronic Structure System (GAMESS) [88,89,90]. The systems for calculations were obtained by extracting AA3266 atoms and those of receptor residues located up to 5 Å from the AA3266 position. The fragmentation setup was done in FACIO [91]. The FMO calculation was a single point energy computation at the MP2/6-31 G*/PCM level, with pair interaction energy decomposition analysis (PIEDA) [92]. The FMO results were analysed separately for Tyr-D-Ala-Gly-Phe- and -NH-NH-Z-D-Trp fragments (at both receptors). Total pair interaction energies (PIE) were calculated as a sum of PIE for a given fragment and the receptor residues. PIE value consists of the following contributions to total energy: E_es_—electrostatic, E_ex_—exchange repulsion, E_ct_—charge-transfer, E_disp_—dispersion, E_solv_—the Gibbs solvation energy. The index %E_es+ct_ describing how much polar an interaction is was calculated following the proposition by Śliwa et al. [93], according to the equation:

$$\%E_{es+ct}=\frac{\left|E_{es}\right|+\left|E_{ct}\right|}{\left|E_{es}\right|+\left|E_{ct}\right|\;+\;\left|E_{disp}\right|}\times 100\%$$

## Figures and Tables

**Table 1:** Cytotoxic selectivity of AA3266 and reference compounds. Average (±S.D.) readout reductions (compared to control) in cell counting and MTT assays, after incubation with 100 µM.

Cmpd	Description	Cell Counting				MTT			
		Readout Reduction (at 100 µM) [Percentage Points] ^1^			Selectivity ^2^	Readout Reduction (at 100 µM) [Percentage Points] ^1^			Selectivity ^2^
		Melanoma ^3^	All Cancers ^4^	Normal Cells ^5^		Melanoma ^3^	All Cancers ^4^	Normal Cells ^5^	
AA3266 This paper and [29]	potent µOR and δOR agonist; moderate NK1 antagonist	61 ± 16	44 ± 25	18 ± 5	3.4 / 2.5	42 ± 9	44 ± 15	17 ± 13	2.5 / 2.6
AWL3020 [44]	potent δOR agonist; low µOR and NK1R binding	51 ± 26	47 ± 19	33 ± 6	1.6 / 1.4	38 ± 21	44 ± 20	43 ± 6	0.9 / 1.0
Aprepitant [29,45]	potent NK1-antagonist	36 ± 3	37 ± 2	32 ± 4	1.1 / 1.2	40 ± 16	38 ± 12	28 ± 5	1.4 / 1.3

^1^ Readout reduction expressed in percentage points, obtained by subtracting the normalized readout for a particular compound from the control value (100%), ^2^ selectivity is calculated by dividing the reduction observed in either the melanoma group or all cancers group by the reduction observed in normal cells group, ^3^ MeW164, MeW155 and MeW151 cell lines, ^4^ MeW164, MeW155, MeW151, E14, T24 cell lines, ^5^ Fib9, FlW180 and FlWp95 cell lines.

**Table 2:** Interaction energies (FMO PIEDA, kcal/mol) of the Tyr-d-Ala-Gly-Phe-fragment binding modes at MOR.

		PIEDA							Contribution of Single Residues				
Pose	Populated [%]	PIE ^1^	E_es_^2^	E_ex_^3^	E_ct_^4^	E_disp_^5^	E_solv_^6^	%E_es+ct_^7^	Tyr1	D-Ala2	Gly3	Phe4	Tyr1—Asp147 Interaction ^8^
**MOR-1**	72	−226.3	−237.4	53.2	−24.5	−36.9	19.3	88	−188.1	−8.2	−20.1	−10.0	−108.2 (48%)
**MOR-2**	10	−160.1	−173.9	40.1	−22.6	−35.4	31.6	85	−126.7	−19.5	-	−13.9	−91.4 (57%)
**MOR-3**	5	−171.3	−185.7	61.4	−27.5	−46.7	27.2	82	−104.0	−15.8	−18.1	−33.3	−70.7 (41%)
**MOR-4**	5	−223.4	−251.9	80.8	−30.8	−51.5	30.0	85	−139.5	−30.6	−20.6	−32.8	−91.0 (41%)

^1^ PIE—pair interaction energy (total) between the receptor and the Tyr-d-Ala-Gly-Phe-fragment, ^2^ E_es_—electrostatic contribution to PIE, ^3^ E_ex_—exchange repulsion contribution to PIE, ^4^ E_ct_—charge-transfer contribution to PIE, ^5^ E_disp_—dispersion contribution to PIE, ^6^ E_solv_—the Gibbs solvation energy, ^7^ %E_es+ct_—percentage share of polar character of interaction (see Methods for definition), ^8^ the number in parentheses is the share of Tyr1 ··· Asp147 PIE in the total PIE.

**Table 3:** Description of representative structures for clusters related to -NH-NH<-Z-d-Trp part.

Title	Title	Intermolecular Contacts		
	Populated [%]	D-Trp	Z	*N*′-Acylhydrazide
**MOR-a**	4	Lys303, Trp318	Arg211 (hydrophobic)	Asp216
**MOR-b**	6	In vicinity of d-Ala2 (Figure S7), Lys233, Val300	to the solvent	Thr218, Leu219 (bb ^1^)
**MOR-c**	11	Ser62 (bb-H-bond), Pro63	Gly60, Thr61	Asn127, Gln124 (bb)
**MOR-d**	4	Arg211 (hydrophobic)	Lys303	Asp216, Glu229, Glu310
**MOR-e**	6	Trp225, Trp228	to the solvent	Thr218
**MOR-f**	11	Lys233 (hydrophobic)	Arg211 (hydrophobic)	-
**MOR-g**	6	Trp318, His319, Tyr128	to the solvent	-
**MOR-h**	3	Thr60, Tyr128	Arg211 (hydrophobic)	-
**MOR-i**	2	Trp318, His319, Gln127, Asn127, Tyr128	Arg211 (hydrophobic)	Asp216

^1^ bb—backbone.

**Table 4:** Interaction energies (FMO PIEDA, kcal/mol) of the -NH-NH<-Z-D-Trp fragment binding modes at MOR.

		PIEDA						
	Populated [%]	PIE ^1^	E_es_^2^	E_ex_^3^	E_ct_^4^	E_disp_^5^	E_solv_^6^	%E_es+ct_^7^
**MOR-a**	4	−55.2	−42.6	11.4	−6.9	−20.5	3.5	71
**MOR-b**	6	−62.4	−43.0	26.0	−12.1	−21.2	−12.1	72
**MOR-c**	11	−63.4	−36.3	26.7	−14.1	−37.1	−2.7	58
**MOR-d**	4	−66.3	−24.2	11.9	−4.1	−14.8	−35.1	66
**MOR-e**	6	−81.8	−41.9	31.6	−12.1	−23.9	−35.6	69
**MOR-f**	11	−56.1	−29.5	11.1	−5.0	−14.0	−18.7	71
**MOR-g**	6	−38.4	−28.3	9.5	−7.8	−20.0	8.1	64
**MOR-h**	3	−92.7	−60.7	20.8	−11.4	−28.0	−13.5	72
**MOR-i**	2	−66.7	−44.1	13.2	−9.4	−17.9	−8.5	75

^1^ PIE—pair interaction energy (total) between the receptor and the -NH-NH<-Z-D-Trp fragment, ^2^ E_es_—electrostatic contribution to PIE, ^3^ E_ex_—exchange repulsion contribution to PIE, ^4^ E_ct_—charge-transfer contribution to PIE, ^5^ E_disp_—dispersion contribution to PIE, ^6^ E_solv_—the Gibbs solvation energy, ^7^ %E_es+ct_—percentage share of polar character of interaction (see Methods for definition).

**Table 5:** Interaction energies (FMO PIEDA, kcal/mol) of the -NH-NH<-Z-d-Trp fragment binding modes at NK1R.

		PIEDA						
	Populated [%]	PIE ^1^	E_es_^2^	E_ex_^3^	E_ct_^4^	E_disp_^5^	E_solv_^6^	%E_es+ct_^7^
**NK1-a**	60	−78.8	−65.7	61.3	−19.8	−51.1	−3.5	63
**NK1-b**	13	−68.7	−44.1	33.2	−12.0	−45.4	−0.4	55
**NK1-c**	3	−58.2	−23.7	23.1	−14.0	−40.4	−3.3	48
**NK1-d**	4	−49.8	−20.6	30.6	−13.5	−44.4	−1.9	43

^1^ PIE—pair interaction energy (total) between the receptor and the -NH-NH<-Z- d-Trp fragment, ^2^ E_es_—electrostatic contribution to PIE, ^3^ E_ex_—exchange repulsion contribution to PIE, ^4^ E_ct_—charge-transfer contribution to PIE, ^5^ E_disp_—dispersion contribution to PIE, ^6^ E_solv_—the Gibbs solvation energy, ^7^ %E_es+ct_—percentage share of polar character of interaction (see Methods for definition).

**Table 6:** Description of representative structures for clusters related to Tyr- d-Ala-Gly-Phe-fragment position in NK1R.

	Populated [%]	Tyr1 Amine	Notes
**NK1-1**	4	To the solvent	By ECL2
**NK1-2**	17	Glu193	By ECL2, Tyr^1^ ring by ECL3
**NK1-3**	2	Asn189	By ECL2
**NK1-4**	7	Glu193	By ECL2
**NK1-5**	4	Glu193	By ECL2
**NK1-6**	9	Glu193	By ECL2, Tyr^1^ ring stack to Phe267
**NK1-7**	5	Glu193	By ECL2, Tyr^1^ ring by ECL3
**NK1-8**	14	Glu193	ByECL2, Tyr^1^ ring towards the receptor interior, stacking with His197, intramolecular H-bond between carbohydrazide and phenol of Tyr^1^
**NK1-9**	3	Glu193	By ECL2, Tyr^1^ H-bond to Tyr287
**NK1-10**	13	Glu193	By ECL2, Tyr^1^ ring directed towards the receptor interior

**Table 7:** Interaction energies (FMO PIEDA, kcal/mol) of the Tyr-d-Ala-Gly-Phe-fragment binding modes at MOR.

		PIEDA							Contribution of Single Residues			
	Populated [%]	PIE ^1^	E_es_^2^	E_ex_^3^	E_ct_^4^	E_disp_^5^	E_solv_^6^	%E_es+ct_^7^	Tyr1	D-Ala2	Gly3	Phe4
**NK1-1**	4	−115.8	−116.0	40.4	−21.3	−56.8	37.8	71	−13.8	−5.5	−5.1	−22.0
**NK1-2**	17	−113.2	−159.5	29.4	−18.6	−21.9	57.5	89	−71.6	−6.4	−8.4	−26.8
**NK1-3**	2	−111.2	−165.6	33.5	−16.6	−19.2	56.7	90	−81.9	−12.4	−3.4	−13.6
**NK1-4**	7	−208.5	−214.6	88.4	−38.3	−77.3	33.2	77	−111.3	−17.6	−2.1	−9.2
**NK1-5**	4	−153.1	−201.6	59.0	−23.7	−26.5	39.7	89	−86.7	−26.6	−26.9	−12.9
**NK1-6**	9	−148.8	−193.1	53.7	−22.5	−32.0	45.0	87	−115.3	−7.5	−12.2	−13.8
**NK1-7**	5	−123.2	−167.4	32.5	−17.3	−21.8	50.8	89	−89.1	−13.8	−	−20.3
**NK1-8**	14	−125.0	−162.3	47.6	−20.8	−30.0	40.5	86	−77.6	−20.5	−8.0	−13.0
**NK1-9**	3	−69.2	−75.6	12.7	−6.8	−22.3	22.8	79	−47.1	−4.6	−2.6	−15.1
**NK1-10**	13	−176.2	−216.7	49.5	−19.6	−23.0	33.6	91	−102.1	−22.6	−26.5	−24.9

^1^ PIE—pair interaction energy (total) between the receptor and the Tyr-d-Ala-Gly-Phe-fragment, ^2^ E_es_—electrostatic contribution to PIE, ^3^ E_ex_—exchange repulsion contribution to PIE, ^4^ E_ct_—charge-transfer contribution to PIE, ^5^ E_disp_—dispersion contribution to PIE, ^6^ E_solv_—the Gibbs solvation energy, ^7^ %E_es+ct_—percentage share of polar character of interaction (see Methods for definition).

## Supplementary Materials

Supplementary materials can be found at https://www.mdpi.com/1422-0067/21/20/7738/s1.

## Abbreviations

%E_es+ct_	Percentage share of polar character of interaction according to PIEDA analysis
%MPE	Percent of the maximal possible effect
ACN	Acetonitrile
AUC	Area under the curve
CCK	Cholecystokinin
DALDA	[d-Arg^2^, Lys^4^]-Dermorphin-(1-4)-amide
DAMGO	[d-Ala^2^, N-Me-Phe^4^, Gly^5^-ol]-Enkephalin
DCC	*N*,*N*′-dicyclohexylcarbodiimide
DCU	*N*,*N*′-dicyclohexylourea
Dmt	2′,6′-Dimethyl-l-tyrosine
DMF	Dimethylformamide
DOR	δ-Opioid receptor
E_ct_	Change-transfer contribution to PIE
E_disp_	Dispersion contribution to PIE
E_ex_	Exchange repulsion contribution to PIE
E_es_	Electrostatic contribution to PIE
ESI-MS	Electrospray-ionization mass spectrometry
E_solv_	Gibbs solution energy
FA	Formic acid
FMO	Fragment molecular orbital
GAMESS	General Atomic and Molecular Electronic Structure System
GTPγS	Guanosine 5′-O-[γ-thio]triphosphate
HOSu	*N*-Hydroxysuccinimide
HPLC	High performance liquid chromatography
IC_50_	Half-maximal inhibitory concentration
Ki	Inhibition constant
Ki-67	Ki-67 protein
MD	Molecular dynamics
MEM	Minimal Essential Medium
MF	Morphine
MOR	μ-Opioid receptor
MTT	3-(4,5-Dimethylthiazol-2-yl)-2,5-diphenyltetrazolium bromide
NK1R	Neurokinin-1 receptor
OPM	Orientation of Proteins in Membrane Database
PIE	Pair interaction energy
PIEDA	Pair interaction energy decomposition analysis
POPC	Phosphatidylcholine
R	Correlation coefficient
RMSD	Root mean square deviation
RP-HPLC	Reversed-phase high performance liquid chromatography
SP	Substance P
TFA	Trifluoroacetic acid
TLC	Thin layer chromatography
TM	Transmembrane helix
TMG	1,1,3,3-Tetramethylguanidine
VGCC	Voltage gated calcium channel
