# Supplementary material for: In Vivo, In Vitro and In Silico Studies of the Hybrid Compound AA3266, an Opioid Agonist/NK1R Antagonist with Selective Cytotoxicity
Source: Int J Mol Sci. 2020 Oct 19;21(20):7738. doi: 10.3390/ijms21207738 (PMC7588979; doi:10.3390/ijms21207738)
Supplement: Supplementary file 1 [file ijms-21-07738-s001.pdf]

Supplementary Materials to:

In vivo, in vitro and in silico studies on AA3266, an opioid peptide with selective cytotoxicity

By:

Joanna Matalińska [1,*], Piotr F.J. Lipiński [1], Piotr Kosson [2], Katarzyna Kosińska [1], and Aleksandra Misicka [1,3,*]

[1] Department of Neuropeptides, Mossakowski Medical Research Centre Polish Academy of Sciences, Pawińskiego 5, 02-106 Warsaw, Poland; jmatalinska@imdik.pan.pl (J.M.); plipinski@imdik.pan.pl (P.F.J.L.); kkosinska@imdik.pan.pl (K.K.); misicka@chem.uw.edu.pl (A.M.)

[2] Toxicology Research Laboratory, Mossakowski Medical Research Centre Polish Academy of Sciences, Pawińskiego 5, 02-106 Warsaw, Poland; pkosson@imdik.pan.pl

[3] Faculty of Chemistry, University of Warsaw, Pasteura 1, 02-093 Warsaw, Poland; misicka@chem.uw.edu.pl

* Correspondence: jmatalinska@imdik.pan.pl (J.M.); misicka@chem.uw.edu.pl (A.M.).

Table of contents:

Figure S1. RMSD plot of the protein in the simulations of AA3266/MOR complex.
Figure S2. RMSD plot of the Tyr-D-Ala-Gly-Phe- fragment in the simulations of AA3266/MOR complex.
Figure S3. RMSD plot of the -NH-NH<-Z-D-Trp fragment in the simulations of AA3266/MOR complex.
Figure S4. Superposition of **MOR-1** binding pose and experimental position of DAMGO in 6DDF PDB structure.
Figure S5. Distance between Tyr^1 amine nitrogen in AA3266 and Asp147 (CG) in MD simulations of AA3266/MOR complex.
Figure S6. Distance between Tyr^1 phenol oxygen in AA3266 and His297 (NE) in MD simulations of AA3266/MOR complex.
Figure S7. Conformation of AA3266 in MOR-b binding pose.
Figure S8. RMSD plot of the protein in the simulations of AA3266/NK1R complex.
Figure S9. RMSD plot of the -NH-NH<-Z-D-Trp fragment in the simulations of AA3266/NK1R complex.
Figure S10. RMSD plot of the Tyr-D-Ala-Gly-Phe- fragment in the simulations of AA3266/NK1R complex.
Figure S11. Distance between carbamate oxygen in AA3266 and Gln165 (NE) in MD simulations of AA3266/NK1R complex.
Figure S12. Distance between acyl oxygen (of the opioid side) of the *N'*-acylhydrazide in AA3266 and Asn109 (ND) in MD simulations of AA3266/NK1R complex.
Figure S13. Distance between hydrogen (of the D-Trp side) of the *N'*-acylhydrazide in AA3266 and Asn109 (OD) in MD simulations of AA3266/NK1R complex.
Figure S14. Distance between Tyr1 amine in AA3266 and Glu193 (CD) in MD simulations of AA3266/NK1R complex.

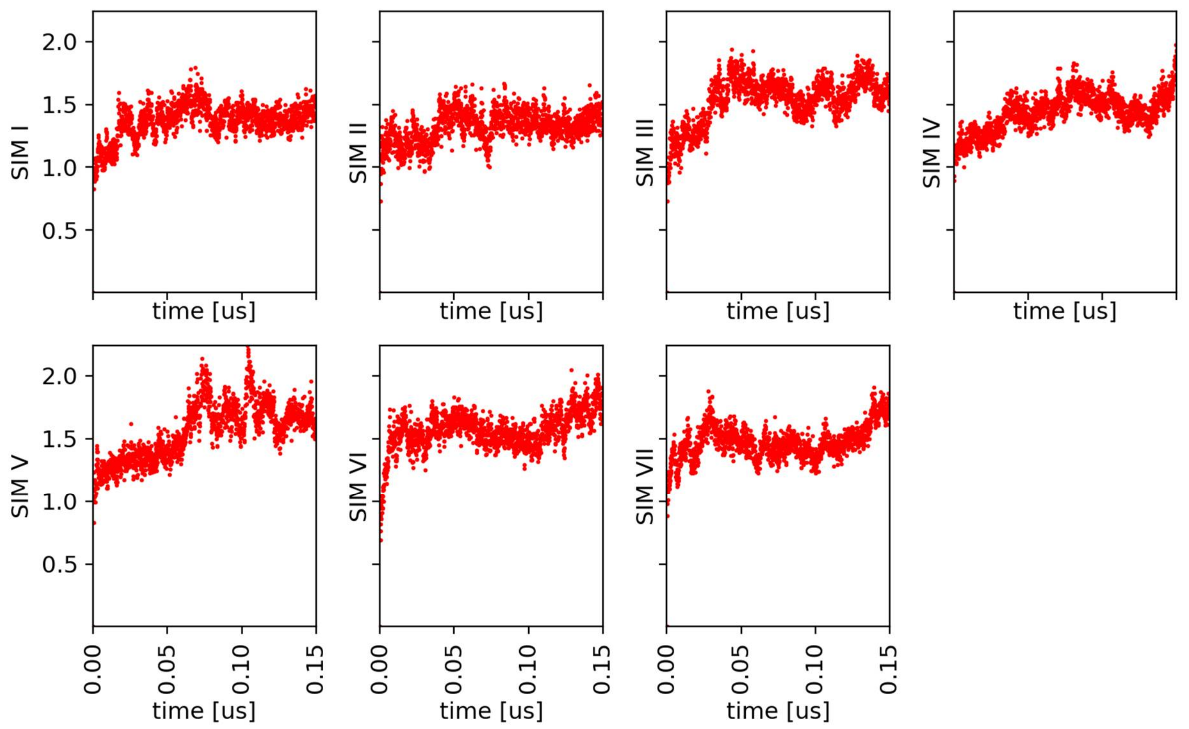

Figure S1. RMSD plot of the protein in the simulations of AA3266/MOR complexes.

RMSD is given in Angstrems.

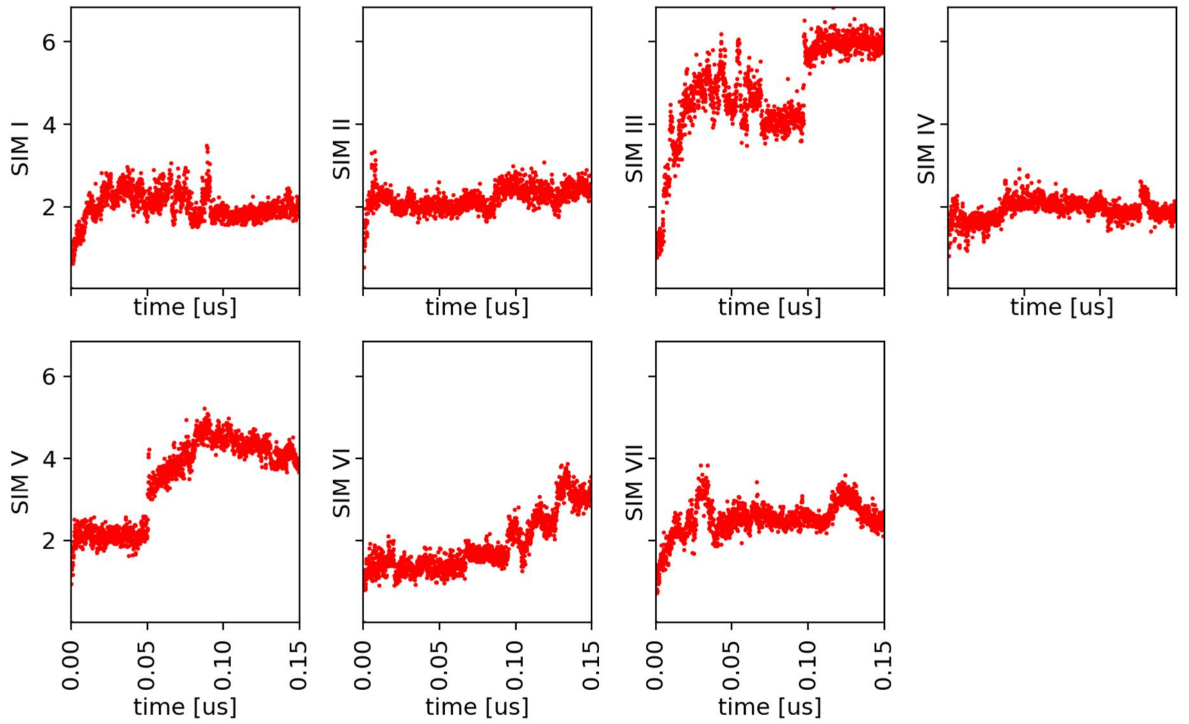

Figure S2. RMSD plot of the Tyr-D-Ala-Gly-Phe- fragment in the simulations of AA3266/MOR complexes.

RMSD is given in Angstrems.

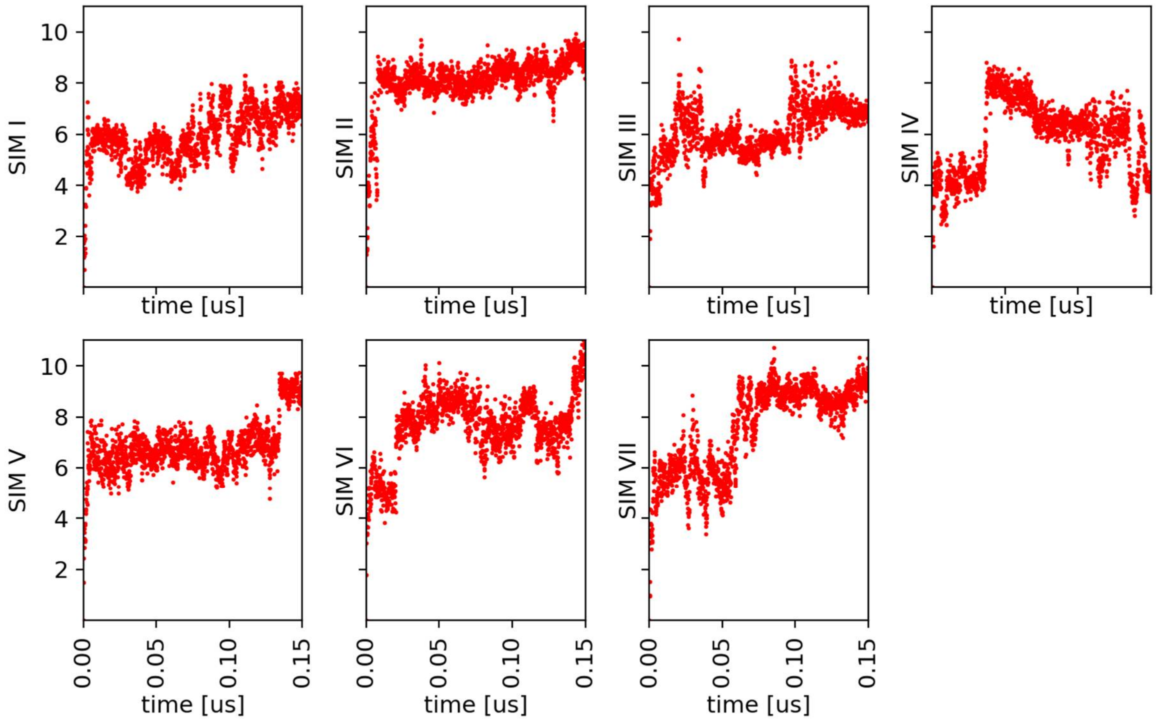

Figure S3. RMSD plot of the -NH-NH<-Z-D-Trp fragment in the simulations of AA3266/MOR complexes.

RMSD is given in Angstrems.

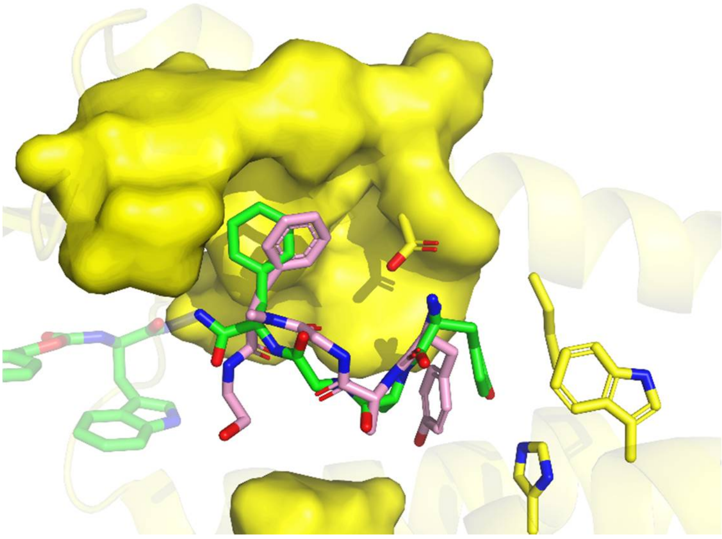

Figure S4. Superposition of **MOR-1** binding pose and experimental position of DAMGO in 6DDF PDB structure.

AA3266 is in green, DAMGO in pink. The receptor is shown in a simplified manner (yellow).

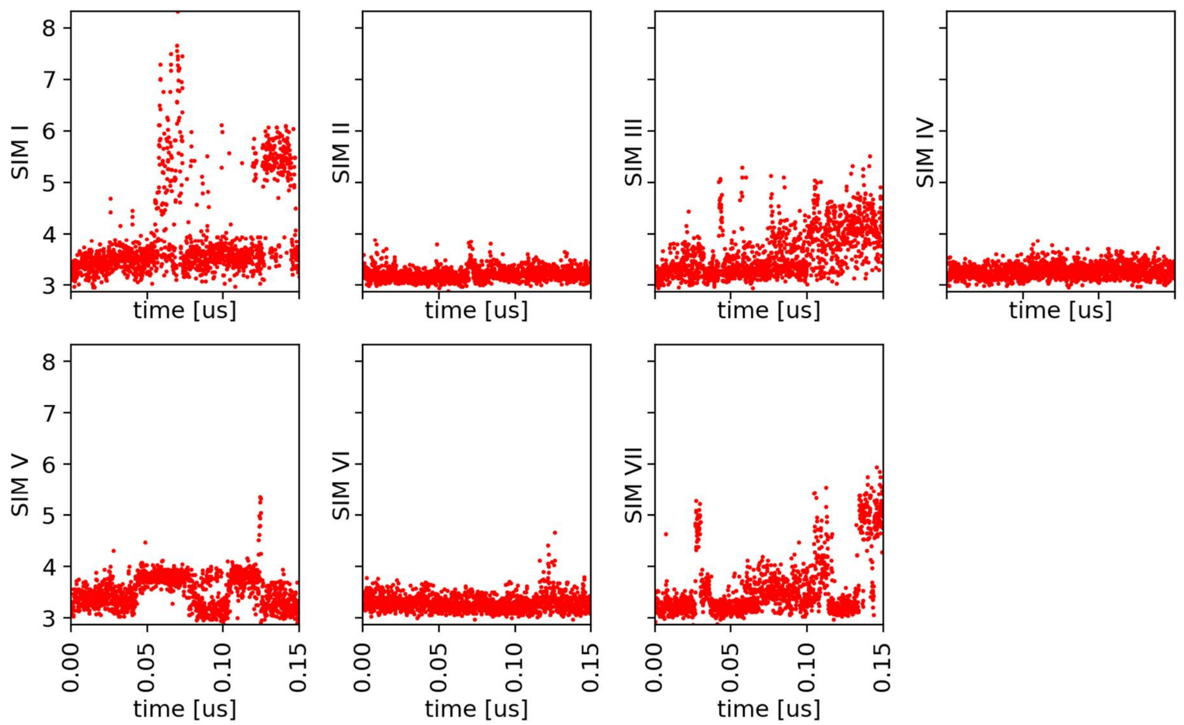

Figure S5. Distance between Tyr[1] amine nitrogen in AA3266 and Asp147 (CG) in MD simulations of AA3266/MOR complex.

Distance is given in Angstrems.

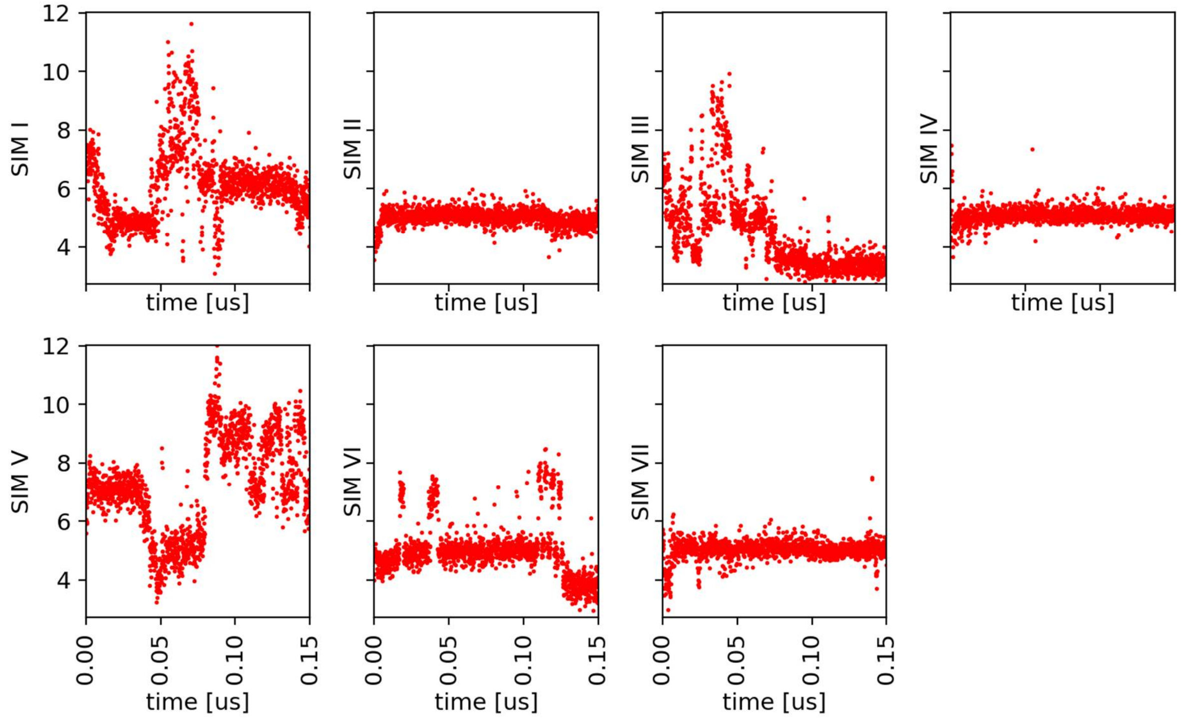

Figure S6. Distance between Tyr[1] phenol oxygen in AA3266 and His297 (NE) in MD simulations of AA3266/MOR complex.

Distance is given in Angstrems.

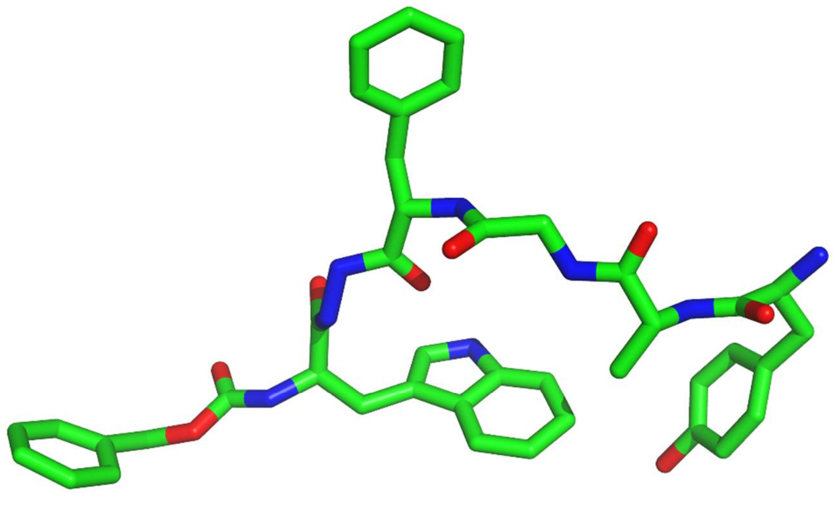

Figure S7. Conformation of AA3266 in **MOR-b** binding pose.

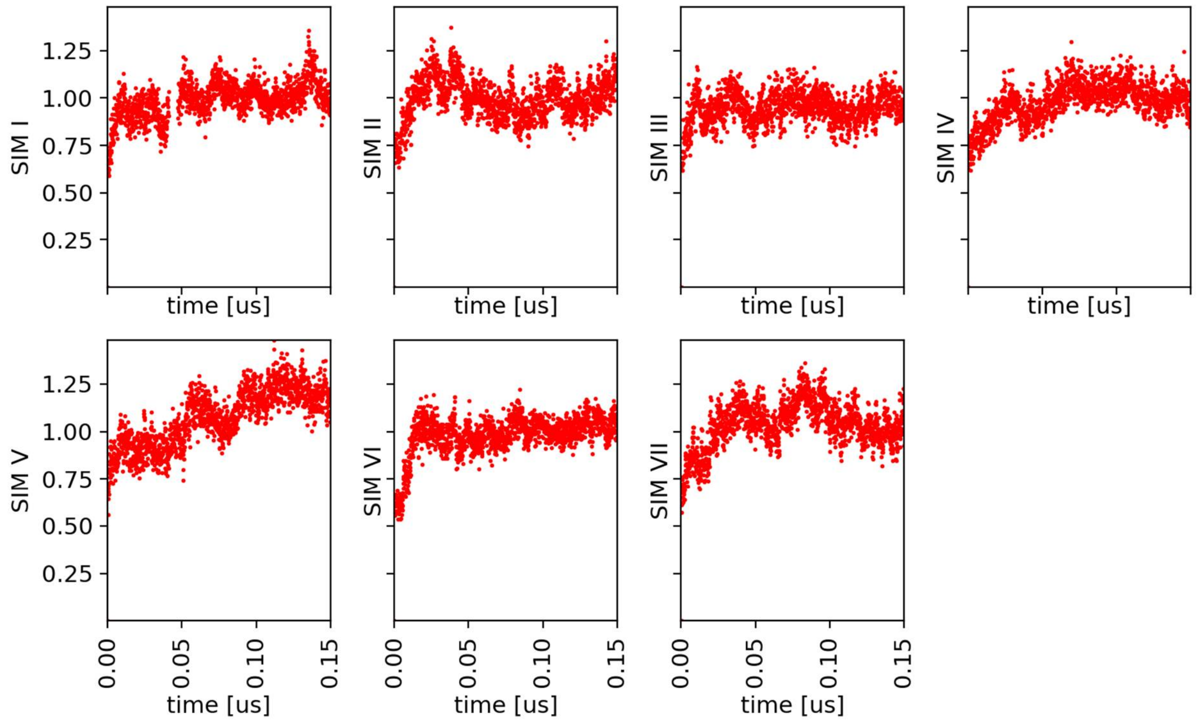

Figure S8. RMSD plot of the protein in the simulations of AA3266/NK1R complexes.

RMSD is given in Angstrems.

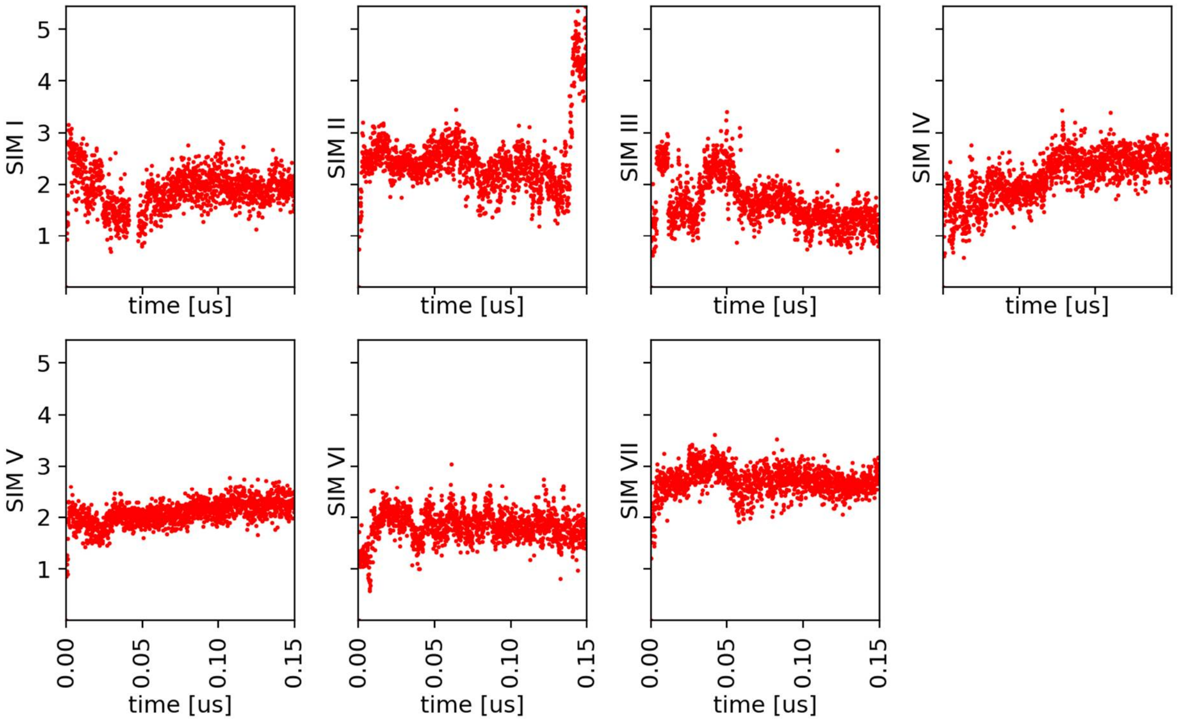

Figure S9. RMSD plot of the -NH-NH<-Z-D-Trp fragment in the simulations of AA3266/NK1R complexes.

RMSD is given in Angstrems.

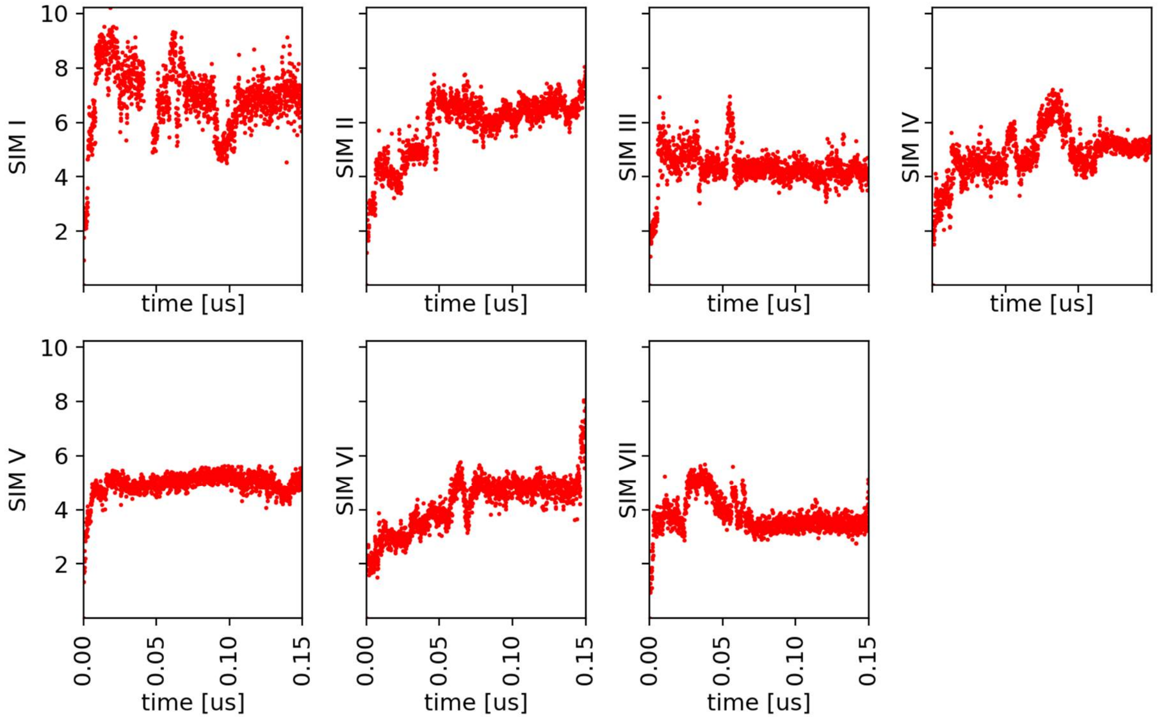

Figure S10. RMSD plot of the Tyr-D-Ala-Gly-Phe- fragment in the simulations of AA3266/NK1R complexes.

RMSD is given in Angstrems.

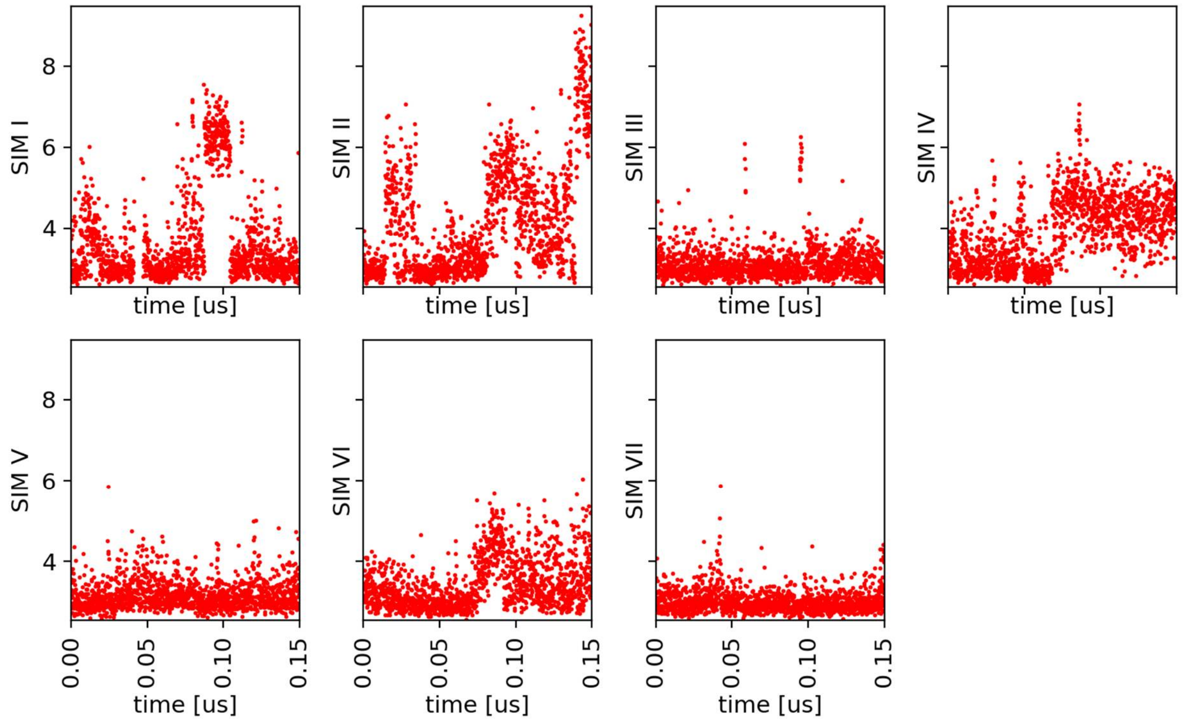

Figure S11. Distance between carbamate oxygen in AA3266 and Gln165 (NE) in MD simulations of AA3266/NK1R complex.

Distance is given in Angstrems.

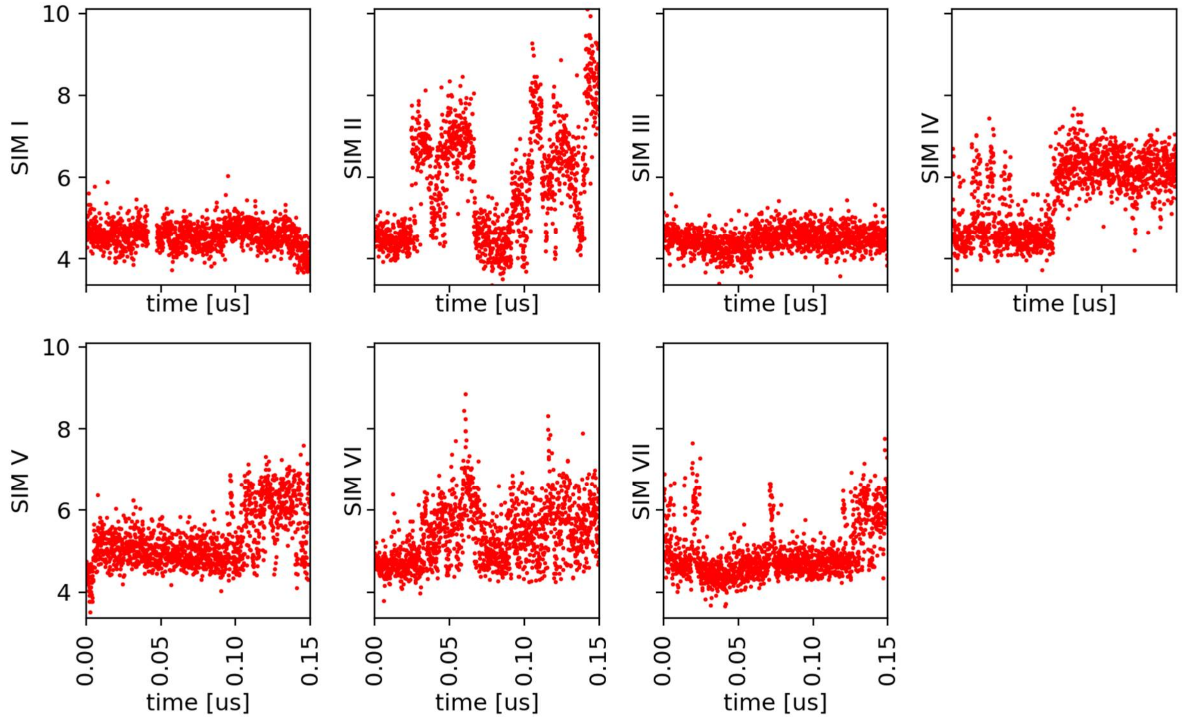

Figure S12. Distance between acyl oxygen (of the opioid side) of the *N'*-acylhydrazide in AA3266 and Asn109 (ND) in MD simulations of AA3266/NK1R complex.

Distance is given in Angstrems.

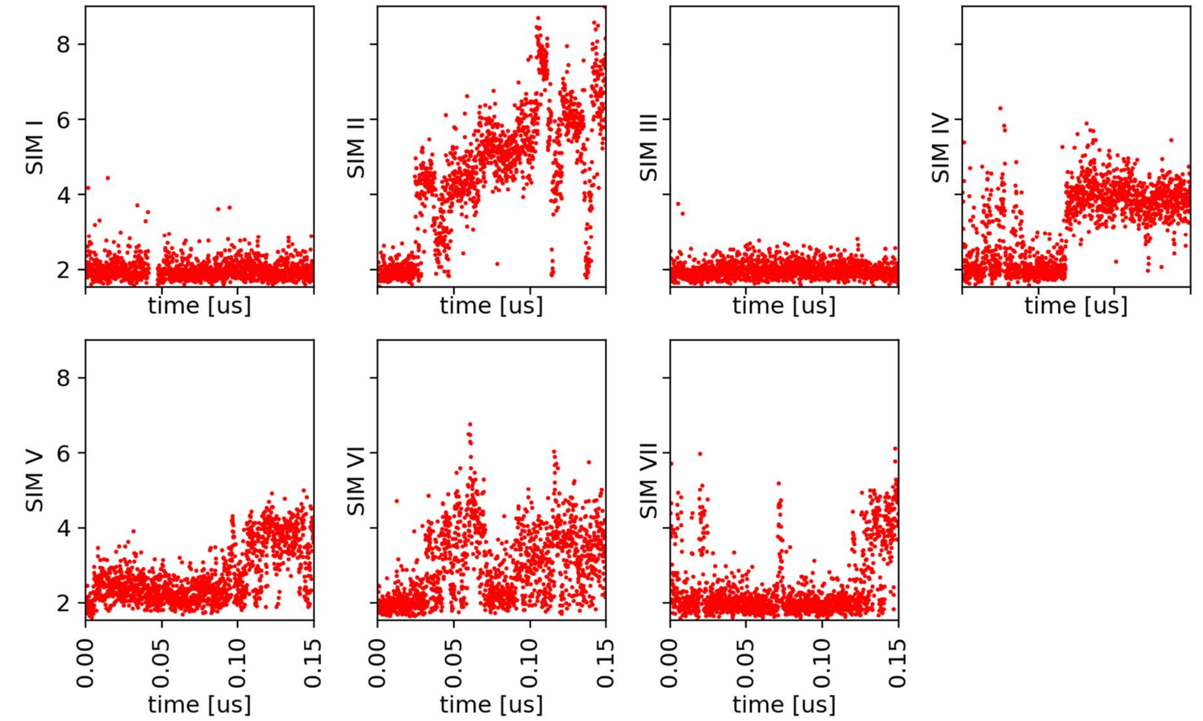

Figure S13. Distance between hydrogen (of the D-Trp side) of the *N'*-acylhydrazide in AA3266 and Asn109 (OD) in MD simulations of AA3266/NK1R complex.

Distance is given in Angstrems.

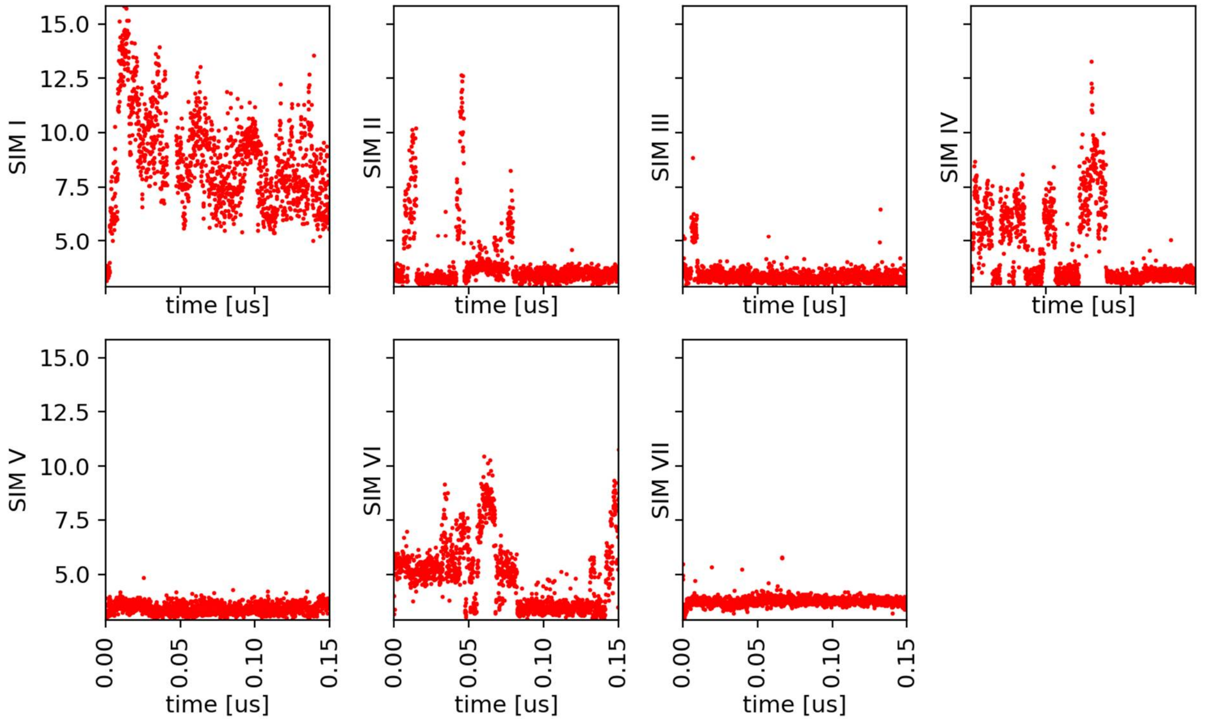

Figure S14. Distance between Tyr1 amine in AA3266 and Glu193 (CD) in MD simulations of AA3266/NK1R complex.

Distance is given in Angstrems.
